# Sodium nitrate protects against metabolic syndrome by sialin-mediated macrophage rebalance

**DOI:** 10.1038/s41392-025-02418-1

**Published:** 2025-10-02

**Authors:** Shaorong Li, Yaning Wang, Zihan Zhang, Haozhe Xu, Songyue Wu, Hua Jin, Xiaotong Han, Ying Liu, Xin Wen, Yi Wu, Zhongtao Zhang, Lei Hu, Liang Hu, Chunmei Zhang, Jinsong Wang, Renhong Yan, Mo Chen, Guozhi Xiao, Guangyong Sun, Dong Zhang, Songlin Wang

**Affiliations:** 1 Salivary Gland Disease Center and Beijing Key Laboratory of Tooth Regeneration and Function Reconstruction, Beijing Laboratory of Oral Health and School of Stomatology, Capital Medical University, Beijing, China; 2 Immunology Research Center for Oral and Systemic Health, Beijing Friendship Hospital, Capital Medical University, Beijing, China; 3 Department of Endodontics, School of Stomatology, Capital Medical University, Beijing, China; 4 Medical Research Center, Beijing Institute of Respiratory Medicine and Beijing Chao-Yang Hospital, Capital Medical University, Beijing, China; 5 Department of Biochemistry, School of Medicine, Southern University of Science and Technology, Shenzhen, Guangdong Province China; 6 Key University Laboratory of Metabolism and Health of Guangdong, SUSTech Homeostatic Medicine Institute, Institute for Biological Electron Microscopy, Southern University of Science and Technology, Shenzhen, Guangdong Province China; 7 Department of Pharmacology, Joint Laboratory of Guangdong–Hong Kong Universities for Vascular Homeostasis and Diseases, School of Medicine and SUSTech Homeostatic Medicine Institute (SHMI), Southern University of Science and Technology, Shenzhen, Guangdong Province China; 8 Department of Laboratory Animal Sciences, School of Basic Medical Sciences, Capital Medical University, Beijing, China; 9 Laboratory Animal Center, Capital Medical University, Beijing, China; 10 Department of General Surgery, National Clinical Research Center for Digestive Diseases, Beijing Friendship Hospital, Capital Medical University, Beijing, China; 11 Department of Oral Maxillofacial Surgery, School of Stomatology, Capital Medical University, Beijing, China; 12 Department of Biochemistry and Molecular Biology, School of Basic Medical Sciences, Capital Medical University, Beijing, China; 13 Department of Biochemistry, School of Medicine, Shenzhen Key Laboratory of Cell Microenvironment, Guangdong Provincial Key Laboratory of Cell Microenvironment and Disease Research, Southern University of Science and Technology, Shenzhen, Guangdong Province China; 14 Laboratory for Oral and General Health Integration and Translation, Beijing Tiantan Hospital, Capital Medical University, Beijing, China; 15 Laboratory of Homeostatic Medicine, School of Medicine, Southern University of Science and Technology, Shenzhen, Guangdong Province China

**Keywords:** Metabolic disorders, Innate immune cells, Immunological disorders, Innate immunity

## Abstract

Metabolic syndrome, characterized by metabolic dysfunction-associated steatotic liver disease (MASLD) and type 2 diabetes mellitus (T2DM), poses a significant threat to patients’ health worldwide; however, efficient treatment is currently unavailable. Here, we show that oral administration of sodium nitrate (NaNO_3_) greatly attenuates the development and advancement of MASLD-like and T2DM-like phenotypes in mice induced by choline-deficient high-fat, western, or methionine/choline-deficient diet. NaNO_3_ attenuates metabolic turbulence by rebalancing CD206^+^/CD11C^+^ polarization (anti-inflammatory/pro-inflammatory) and the function of bone marrow-derived macrophages (MoMFs). Using metabolic disorder animal models and bone marrow-reconstituted mice with mutated gene function in *Slc17a5*, which encodes sialin, we demonstrate that NaNO_3_ protects against metabolic disorders through the actions of sialin in MoMFs. NaNO_3_ can directly regulate MoMFs polarization and function in vitro and in mice, in which nitric oxide production from oral and enteral symbiotic bacteria is essentially abolished. At the molecular level, sialin, via the inhibition of the key transcription factor Rel, inhibits cathepsin L (CtsL) expression and thereby activates the Nrf2 pathway to modulate macrophage homeostasis and ameliorate metabolic abnormalities. Interestingly, the sialin-CtsL-Nrf2 pathway is downregulated in human macrophages from metabolic dysfunction-associated steatohepatitis (MASH) patients. Overall, we demonstrate the prophylactic and therapeutic effects of NaNO_3_ on metabolic syndrome and reveal a new macrophage rebalancing strategy involving NaNO_3_ through a novel sialin pathway. Our research indicates that NaNO_3_ may be a pharmaceutical agent for managing and alleviating metabolic turbulence in humans.

## Introduction

Metabolic disorders, a major category of diseases, encompassing metabolic dysfunction-associated steatotic liver disease (MASLD) and type 2 diabetes mellitus (T2DM), represent a spectrum of liver and systemic metabolic turbulences involving lipids, glucose, and multiple systemic dysregulations. This significantly impacts the overall health, both physically and mentally, of affected individuals. Among these conditions, MASLD is recognized as a major global cause of liver disease.^1–3^ MASLD, particularly its histological phenotype, metabolic dysfunction-associated steatohepatitis (MASH), serves as a substitute for nonalcoholic steatohepatitis (NASH) and has the potential to escalate to more serious hepatic complaints, encompassing cirrhosis and hepatocellular carcinoma (HCC).^4^ MASLD and T2DM often coexist and collaborate synergistically, enhancing the likelihood of adverse (both hepatic and extrahepatic) health consequences. T2DM also remains a paramount risk element for the accelerated advancement of MASLD toward MASH, progressive fibrosis, or cirrhosis. Failure to promptly rectify these metabolic turbulences can lead to a cascade of complications involving the heart, brain, and other vital systems, posing a threat to life.

Immune cells of the liver, particularly hepatic macrophages, function as key factors in the progression of MASLD/MASH or other metabolic disorders. According to the previous research, hepatic macrophages encompass diverse cellular subsets. Among these subsets, bone marrow monocyte-derived macrophages (MoMFs), infiltrating monocytes, originate from blood monocytes produced by bone marrow resident hematopoietic stem cells.^5,6^ In metabolic disorders, MoMFs can be recruited to differentiate into pro-inflammatory macrophages. The pro-inflammatory macrophages generate multiple inflammation-promoting factors encompassing TNF-α, IL-1β, IL-6, and so on, which not only exacerbate local tissue damage but also contribute to further metabolic disorder progression.^7^ The imbalance between anti-inflammatory MoMFs and pro-inflammatory MoMFs functions significantly in the progression of MASLD/MASH or other metabolic dysfunctions.^7,8^ However, at present, there are no effective therapeutic options for metabolic turbulence available, and it is therefore essential to create innovative approaches for preventing and/or treating these diseases.

Nitrate, a key dietary component, is particularly concentrated in leafy greens such as spinach, lettuce, and beetroot.^9^ Dietary nitrate metabolism, distribution, and excretion involve several key processes in the body. Nitrate can be administered orally, intravenously, or via mucosal absorption (e.g., sublingual). After oral intake, the compound demonstrates strong gastrointestinal absorption and relatively high bioavailability (60–70%). The peak plasma concentration (T_max_) is typically reached within 30–60 min post-ingestion.^10^ In the primary metabolic pathway, nitrate is transformed to nitrite by the mouth and gut microbiota. Subsequently, nitrite is further transformed to nitric oxide (NO) under acidic stimulations or via enzymatic actions. Nitrate ions are widely distributed throughout the body, including in blood and saliva, with the highest concentration found in saliva. The half-life is approximately 5–8 h but may vary depending on individual differences and diet. Nitrate is primarily excreted by the kidneys, with approximately 60–70% being excreted as nitrate ions, while the remainder consists of metabolites.^11,12^ Nitrate is commonly believed to exert its effects mainly via the nitrate‒nitrite‒NO metabolic pathway. Oral or enteral symbiotic microbiota inside the body play an integral role in converting nitrate to nitrite and lead to NO generation.^11,12^ Dietary supplementation with sodium nitrate (NaNO_3_), the most common inorganic form of nitrate, is known to prevent salivary gland damage^13^ and stress-induced gastric injury,^14^ mitigate the risk of hepatic ischemia-reperfusion,^15^ and improve glucolipid metabolism in senescent livers.^16^ In addition, dietary nitrate prevents many features of hepatic steatosis that progressed in mice generated by high-fat nutrition.^17^ However, whether and how nitrate alters the progression of metabolic syndrome, like MASLD and T2DM, remains unclear.

Previous studies suggest that the described function of nitrate cannot be simply dependent on NO produced through the nitrate‒nitrite‒NO sequence. We previously showed that sialin, which is encoded by *Slc17a5*, is important for the transportation of nitrate from serum to salivary gland cells, alongside nitrate recycling and maintenance of nitrite–NO homeostasis.^18^ Sialin is in the lysosomal membrane and functions significantly in the transport of sialic acid.^19^ Sialin is also located in the cytomembrane and is widely distributed in the mouse liver, salivary gland, lung, and many other organs. In the current research, we aimed to investigate whether NaNO_3_, via sialin, attenuates metabolic disorders, especially MASLD and T2DM, by modulating the inflammatory responses of MoMFs to balance immune homeostasis. To examine the hepatoprotective and systemic benefits of dietary NaNO_3_ on metabolic disorders, we used three metabolic disorder animal models. To explore the underlying immune mechanism involving bone marrow MoMFs, we employed a novel *Slc17a5* mutant mouse model with bone marrow reconstitution. To explore the potential effects of NO from oral and enteral symbiotic bacteria on the effects of NaNO_3_ on metabolic disorders, we employed a mouse model treated with antibiotics to eliminate the microbiome. We show that dietary NaNO_3_ greatly attenuates the development and progression of MASLD and T2DM by rebalancing pro- and anti-inflammatory MoMFs via the modulation of a novel sialin-mediated pathway.

## Results

### Oral NaNO_3_ greatly ameliorates MASLD-like phenotypes in mice induced by choline-deficient high-fat diet (CDHFD) or methionine/choline-deficient diet (MCD)

**Fig. 1:**
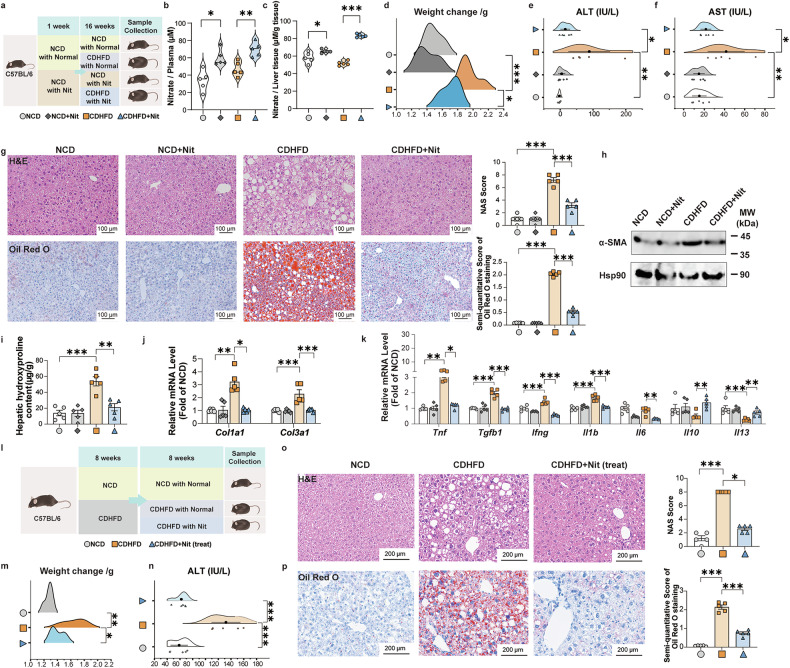
Dietary NaNO_3_ ameliorates lipid metabolic disorders. **a**–**k** C57BL/6 mice received drinking water supplemented with 4 mM NaNO_3_ (Nit) or normal drinking water. After 1 week, half of each group was fed with a choline-deficient high-fat diet (CDHFD), and the others were given a normal control diet (NCD). After 16 weeks, all the mice were sacrificed. **a** Schematic overview of the experiment. **b** Nitrate concentrations in the plasma of the NCD and CDHFD groups (one-way ANOVA with a post hoc test, *P < 0.05, **P < 0.01, *n* = 5, biological replicates). **c** Hepatic nitrate concentrations of the NCD and CDHFD groups (one-way ANOVA with a post hoc test, *P < 0.05, ***P < 0.001, *n* = 5, biological replicates). **d** Body weight changes after the experiment in the NCD and CDHFD groups (one-way ANOVA with a post hoc test, *P < 0.05, ***P < 0.001, *n* = 5, biological replicates). **e** Plasma alanine aminotransferase (ALT) levels in the NCD and CDHFD groups (one-way ANOVA with a post hoc test, *P < 0.05, **P < 0.01, *n* = 5, biological replicates). **f** Plasma aspartate aminotransferase (AST) levels in the NCD and CDHFD groups (one-way ANOVA with a post hoc test, *P < 0.05, **P < 0.01, *n* = 5, biological replicates). **g** Representative H&E-stained mouse liver sections plus a graph showing the MASLD activity score (NAS), representative Oil Red O-stained mouse liver sections plus a graph showing the semiquantitative score. Scale bars: 100 μm (one-way ANOVA with a post hoc test, ***P < 0.001, *n* = 5, biological replicates). **h** Western blot images of α-SMA in the NCD and CDHFD groups (*n* = 5, biological replicates). **i** Hepatic hydroxyproline content of the NCD and CDHFD groups (one-way ANOVA with a post hoc test, **P < 0.01, ***P < 0.001, *n* = 5, biological replicates). **j** Relative mRNA expression of hepatic *Col1a1* and *Col1a3* of the NCD and CDHFD groups (Kruskal‒Wallis test for *Col1a1*, one-way ANOVA with a post hoc test for *Col1a3*, *P < 0.05, **P < 0.01, ***P < 0.001, *n* = 5, biological replicates). **k** Relative mRNA expression of hepatic inflammatory cytokine factors of the NCD and CDHFD groups (Kruskal‒Wallis test for *Tnf* and *Il10*, one-way ANOVA with a post hoc test for others, *P < 0.05, **P < 0.01, ***P < 0.001, *n* = 5, biological replicates). **l**‒**p** Two groups of C57BL/6 mice were fed with CDHFD, and the other group was given NCD for 8 weeks. The mice subsequently received drinking water supplemented with 4 mM NaNO_3_ (Nit) or normal drinking water for another 8 weeks while the diet remained. Then the plasma and livers were collected. **l** Schematic overview of the experiment in NCD- and CDHFD-fed nitrate-treated mice. **m** Changes in body weight after the experiment in NCD- and CDHFD-fed nitrate-treated mice (one-way ANOVA with a post hoc test, *P < 0.05, **P < 0.01, *n* = 5, biological replicates). **n** Plasma ALT levels in NCD- and CDHFD-fed nitrate-treated mice (one-way ANOVA with a post hoc test, ***P < 0.001, *n* = 5, biological replicates). **o**‒**p** Representative H&E-stained mouse liver sections plus a graph showing the NAS and representative Oil Red O-stained mouse liver sections plus a graph showing the semiquantitative score. Scale bars: 200 μm (Kruskal‒Wallis test for NAS, one-way ANOVA with a post hoc test for Oil Red O-stained, *P < 0.05, ***P < 0.001, *n* = 5, biological replicates)

To investigate the potential preventive effect of NaNO_3_ on lipid metabolic disorders, we administered drinking water containing 4 mM NaNO_3_ or normal water to C57BL/6 mice (Fig. 1a). Drawing from our experimental findings (Supplementary Fig. 1a–p), we chosed a concentration of 4 mM NaNO_3_ for this and other indicated experiments in this study. After 1 week, the animals were given a choline-deficient high-fat diet (CDHFD) or a normal control diet (NCD) for 16 weeks, then plasma and liver tissues were obtained upon completion of the study. In both diet groups, the plasma and liver nitrate levels at 17 weeks were markedly greater in the NaNO_3_-treated mice, which had access to normal drinking water (Fig. 1b, c). In the CDHFD-induced model, there was an increase in mouse body weight, and nitrate supplementation decreased body weight (Fig. 1d). Compared with those in the NCD group, the activities of alanine transaminase (ALT) and aspartate transaminase (AST) were markedly greater in CDHFD mice, both of which were downregulated by NaNO_3_ supplement (Fig. 1e, f). The CDHFD led to moderate decreases in water intake and diet intake in mice; however, nitrate water did not cause any marked changes in water or diet intake (Supplementary Fig. 2a, b). Furthermore, NaNO_3_ supplementation significantly decreased the MASLD activity score (NAS) and liver fat accumulation in CDHFD mice (Fig. 1g). The expression of hepatic α-SMA in the NCD, NCD+Nit, CDHFD, and CDHFD+Nit groups was shown in Fig. 1h, and higher levels were observed in the CDHFD groups. NaNO_3_ decreased the high hepatic hydroxyproline content of CDHFD mice (Fig. 1i). The relative hepatic levels of the fibrosis-associated genes *Col1a1* and *Col1a3* were lower in NaNO_3_-treated CDHFD-fed mice (Fig. 1j). NaNO_3_ administration also reduced the expression of inflammation-promoting genes, including *Tnf*, *Tgfb1*, *Ifng*, and *Il1b*, in mice on CDHFD relative to untreated CDHFD-fed controls (Fig. 1k).

We verified these results in another mouse model, i.e., the methionine/choline-deficient diet (MCD)-induced MASLD model (Supplementary Fig. 2c, d). In the MCD-fed mice, dietary 4 mM NaNO_3_ supplementation increased the plasma and liver nitrate concentrations (Supplementary Fig. 2e, f) and decreased the plasma ALT and AST levels (Supplementary Fig. 2g, h), hepatic fat accumulation, and lobular inflammation of the MCD group (Supplementary Fig. 2i–k). The expression of fibrosis-related and pro-inflammatory cytokine genes was also downregulated after NaNO_3_ treatment (Supplementary Fig. 2l, m). The hydroxyproline content in liver tissue was markedly greater in the MCD group, but it was decreased after dietary NaNO_3_ supplementation (Supplementary Fig. 2n).

To assess the therapeutic effects of NaNO_3_ on lipid metabolic disorder, we supplemented NaNO_3_ in the drinking water of mice fed a CDHFD for 8 weeks, followed by CDHFD feeding for another 8 weeks (Fig. 1l). The administration of NaNO_3_ significantly decreased body weight (Fig. 1m), reduced plasma ALT levels (Fig. 1n), and decreased the NAS score and liver fat accumulation (Fig. 1o, p) in mice with CDHFD-induced lipid metabolic disorder.

Taken together, these findings demonstrate that dietary NaNO_3_ supplementation largely attenuates the development and progression of lipid metabolic turbulences in two classical MASLD animal models.

### Oral NaNO_3_ mitigates glucose and lipid metabolic abnormalities and hepatic injury in mice induced by CDHFD and western diet (WD)

**Fig. 2:**
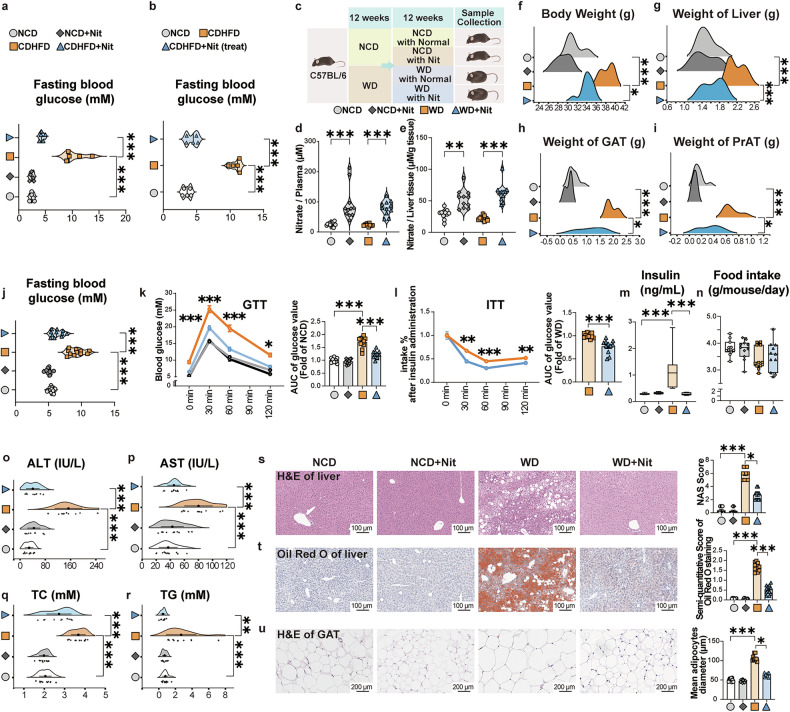
Dietary NaNO_3_ mitigates glucose and other systemic metabolic disorders in mice. **a** Fasting blood glucose levels in the normal control diet (NCD), NCD+Nit, choline-deficient high-fat diet (CDHFD), and CDHFD+Nit prevention groups (one-way ANOVA with a post hoc test, ***P < 0.001, *n* = 5, biological replicates). **b** Fasting blood glucose levels in the NCD-, CDHFD-, and CDHFD+Nit-treated groups (one-way ANOVA with a post hoc test, ***P < 0.001, *n* = 5, biological replicates). **c**–**u** Two groups of mice were fed with western diet (WD), and the others were fed with NCD for 12 weeks. The mice subsequently received drinking water supplemented with 4 mM NaNO_3_ (Nit) or normal drinking water for another 12 weeks while the diet remained. Then the plasma and livers were collected. **c** Schematic overview of the experiment. **d** Nitrate concentrations in plasma of NCD- and WD-fed mice (Kruskal–Wallis test, ***P < 0.001, *n* = 11, biological replicates). **e** Nitrate concentrations in the livers of NCD- and WD-fed mice (Kruskal–Wallis test, **P < 0.01, ***P < 0.001, *n* = 11, biological replicates). **f** The body weight after the experiment in NCD- and WD-fed mice (Kruskal–Wallis test, *P < 0.05, ***P < 0.001, *n* = 11, biological replicates). **g** The weight of livers in NCD- and WD-fed mice (One-way ANOVA with a post hoc test, ***P < 0.001, *n* = 11, biological replicates). **h** The weight of gonadal adipose tissue (GAT) in NCD- and WD-fed mice (Kruskal–Wallis test, *P < 0.05, ***P < 0.001, *n* = 11, biological replicates). **i** The weight of perirenal adipose tissue (PrAT) in NCD- and WD-fed mice (Kruskal–Wallis test, *P < 0.05, ***P < 0.001, *n* = 11, biological replicates). **j** Fasting blood glucose levels in NCD- and WD-fed mice (one-way ANOVA with a post hoc test, ***P < 0.001, *n* = 11, biological replicates). **k** Glucose tolerance test (GTT) results and relative area under the curve values for the NCD- and WD-fed mice (one-way ANOVA with a post hoc test, *P < 0.05, ***P < 0.001, *n* = 11, biological replicates). **l** Insulin tolerance test (ITT) results and relative area under the curve values for the NCD- and WD-fed mice (Kruskal‒Wallis test, **P < 0.01, ***P < 0.001, *n* = 11, biological replicates). **m** The levels of insulin in the NCD- and WD-fed mice (Kruskal‒Wallis test, ***P < 0.001, *n* = 11, biological replicates). **n** Food intake per mouse every day in the NCD and WD groups (Kruskal‒Wallis test, *n* = 11, biological replicates). **o** Plasma alanine aminotransferase (ALT) levels in the NCD and WD groups (one-way ANOVA with a post hoc test, ***P < 0.001, *n* = 11, biological replicates). **p** Plasma aspartate aminotransferase (AST) levels in the NCD and WD groups (one-way ANOVA with a post hoc test, ***P < 0.001, *n* = 11, biological replicates). **q** The levels of plasma total cholesterol (TC) in NCD- and WD-fed mice (one-way ANOVA with a post hoc test, ***P < 0.001, *n* = 11, biological replicates). **r** The levels of plasma triglyceride (TG) in the NCD and WD groups (Kruskal‒Wallis test, **P < 0.01, ***P < 0.001, *n* = 11, biological replicates). **s**–**u** Representative H&E-stained mouse liver sections plus a graph showing the MASLD activity score (NAS) and representative Oil Red O-stained mouse liver sections plus a graph showing the semiquantitative score. Scale bars: 100 μm. Representative H&E-stained mouse GAT plus a graph showing the mean diameter of adipocytes. Scale bars: 200 μm (Kruskal‒Wallis test for NAS and the mean diameter of adipocytes, one-way ANOVA with a post hoc test for Oil Red O staining, *P < 0.05, ***P < 0.001, *n*= 11, biological replicates)

In the CDHFD-induced mouse model described above, the increases in fasting blood glucose levels were largely reversed by NaNO_3_ supplementation (Fig. 2a, b). Next, we supplemented NaNO_3_ in the drinking water of a more “physiological” animal model, which was induced with a western diet (WD) for 12 weeks, followed by continuous WD feeding for another 12 weeks with nitrate water or normal water (Fig. 2c). First, we confirmed that the nitrate concentrations in the plasma and liver tissue were markedly greater after NaNO_3_ supplementation (Fig. 2d, e). WD increased body weight, which was partially decreased by NaNO_3_ (Fig. 2f). Similar results were obtained for the weights of the liver, gonadal adipocyte tissue (GAT), and perirenal adipocyte tissue (PrAT) (Fig. 2g–i). NaNO_3_ additionally attenuated the rise in fasting blood glucose concentrations observed in this experimental model (Fig. 2j). NaNO_3_ improved glucose clearance capacity, as determined by glucose tolerance test (GTT), and increased insulin sensitivity, as demonstrated by insulin tolerance test (ITT), in the WD-fed mouse model (Fig. 2k, l). WD increased the blood insulin level, which was essentially abolished by NaNO_3_ treatment (Fig. 2m). We recorded food intake and found no significant difference between mice with or without NaNO_3_ treatment (Fig. 2n). Similarly, WD increased plasma ALT, AST, total cholesterol (TC), and total triglyceride (TG) levels, which were dramatically downregulated by NaNO_3_ (Fig. 2o–r). Histological analyses of the liver and GAT revealed that NaNO_3_ decreased the NAS score, liver fat accumulation, and mean diameter of adipocytes in WD-fed mice (Fig. 2s–u).

To address the concern of the safety profile of chronic nitrate administration, we established a cohort of animals subjected to prolonged WD feeding with nitrate-supplemented water. The experimental design comprised four groups: NCD with normal water, NCD with nitrate water, WD with normal water, and WD with nitrate water. The mice were administered nitrate in their drinking water and WD at 8 weeks old, and the intervention lasted for 14 months until sample collection. This model successfully induced advanced metabolic pathologies, including hepatic steatosis. The results revealed that nitrate administration had no significant effect on blood glucose, ITT, or GTT under normal conditions (Supplementary Fig. 3a, b). However, it effectively improved obesity (body weight, liver/GAT weight), insulin levels, and insulin resistance in WD-induced metabolic disorder models (Supplementary Fig. 3a–d). For multiorgan safety assessment, comprehensive metabolic analyses were conducted. The results revealed significant improvements in indicators of liver function, including ALT, AST, TC, and TG (Supplementary Fig. 3e–h). The indicators of heart, kidney, and other damage, such as creatine kinase isoenzyme (CK-MB), were reduced by nitrate administration, whereas other indicators, including uric acid (UA), blood urea nitrogen (BUN), creatinine (CRE), triiodothyronine (T3), and thyroxine (T4), were not markedly altered by nitrate supplementation (Supplementary Fig. 3i–m). Furthermore, the results of our rotary test revealed that prolonged nitrate administration produced no significant effect on mouse rotational behavior (Supplementary Fig. 3n–p).

Nitrate concentrations were measured in GAT, PrAT, skeletal muscle, heart, brain, spleen, kidney, large intestine, small intestine, and lung, providing a broader assessment of nitrate distributions across diverse organ systems. As shown in Supplementary Fig. 3q–z, nitrate administration elevated the nitrate concentrations in almost all the organ tissues. Nitrate led to a marked upregulation in levels within the heart, skeletal muscle, kidney, and GAT in mice fed with NCD or WD. We posit that nitrate indeed exerts a global effect, with notable enrichment observed in the liver, plasma, and GAT, as detailed in the research.

### NaNO_3_ modulates CD206^+^/CD11C^+^ polarization and function of bone marrow monocyte-derived macrophages (MoMFs) in vitro and in WD-, CDHFD- or MCD-fed mice

**Fig. 3:**
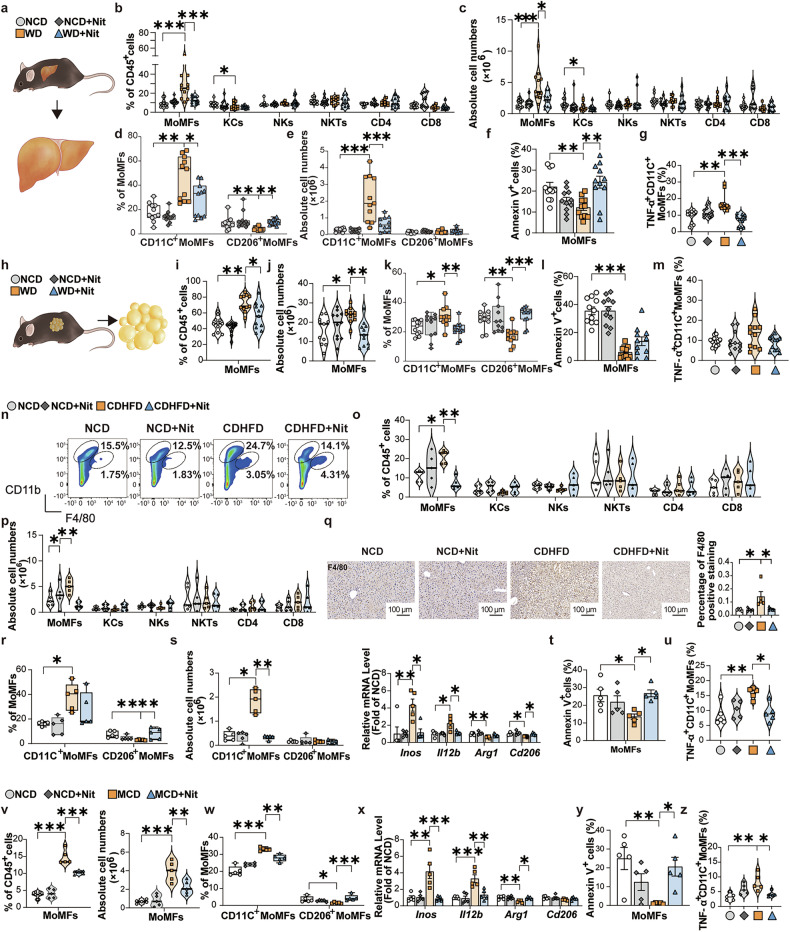
NaNO_3_ modulates MoMFs inflammatory responses and survival in vivo. **a**‒**m** In the first 12 weeks, two groups of C57BL/6 mice were fed with a western diet (WD), and the others were fed with a normal control diet (NCD). The mice subsequently received drinking water supplemented with 4 mM NaNO_3_ (Nit) or normal drinking water for another 12 weeks while the diet remained. Then the plasma and livers were collected (see Fig. 2c). **a** Schematic overview of the experiments for the analysis of liver cells in the NCD and WD groups. **b** Proportions of hepatic bone marrow monocyte-derived macrophages (MoMFs), Kupffer cells (KCs), natural killer cells (NKs), NK T cells (NKTs), and CD4^+^ and CD8^+^ T cells of NCD and WD groups were assessed via flow cytometry plus a graph (one-way ANOVA with a post hoc test for MoMFs, and NKTs, the Kruskal‒Wallis test for KCs, NKs, and CD4^+^ and CD8^+^ T cells, *P < 0.05, ***P < 0.001, *n* = 11, biological replicates). **c** Absolute cell numbers of hepatic MoMFs, KCs, NKs, NKTs, and CD4^+^ and CD8^+^ T cells of NCD and WD groups were determined via flow cytometry (one-way ANOVA with a post hoc test for NKTs, the Kruskal‒Wallis test for MoMFs, KCs, NKs, and CD4^+^ and CD8^+^ T cells, *P < 0.05, ***P < 0.001, *n* = 11, biological replicates). **d** Proportions of hepatic CD11C^+^ MoMFs and CD206^+^ MoMFs of NCD and WD groups (Kruskal‒Wallis test, *P < 0.05, **P < 0.01, *n* = 11, biological replicates). **e** Absolute cell numbers of hepatic CD11C^+^ MoMFs and CD206^+^ MoMFs (Kruskal‒Wallis test for CD206^+^ MoMFs, one-way ANOVA with a post hoc test for CD11C^+^ MoMFs, ***P < 0.001, *n* = 11, biological replicates). **f** Apoptosis (annexin V^+^ cells) of hepatic MoMFs of NCD and WD groups (one-way ANOVA with a post hoc test, **P < 0.01, *n* = 11, biological replicates). **g** Proportion of hepatic TNF-α^+^ CD11C^+^ MoMFs of NCD and WD groups (Kruskal‒Wallis test, **P < 0.01, ***P < 0.001, *n* = 11, biological replicates). **h** Schematic overview of the experiment for the analysis of gonadal adipose cells in the NCD- and WD-fed mice. **i** Proportions of MoMFs in gonadal adipose tissue (GAT) of the NCD and WD groups (Kruskal‒Wallis test, *P < 0.05, **P < 0.01, *n* = 11, biological replicates). **j** Absolute cell numbers of MoMFs in the GAT of the NCD and WD groups (one-way ANOVA with a post hoc test, *P < 0.05, **P < 0.01, *n* = 11, biological replicates). **k** Proportions of CD11C^+^ MoMFs and CD206^+^ MoMFs in the GAT of the NCD and WD groups (Kruskal‒Wallis test, *P < 0.05, **P < 0.01, ***P < 0.001, *n* = 11, biological replicates). **l** Apoptosis (annexin V^+^ cells) of MoMFs in GAT of the NCD and WD groups (one-way ANOVA with a post hoc test, ***P < 0.001, *n* = 11, biological replicates). **m** Proportion of TNF-α^+^ CD11C^+^ MoMFs in the GAT of the NCD and WD groups (one-way ANOVA with a post hoc test, *n* = 11, biological replicates). **n**–**u** C57BL/6 mice received 4 mM NaNO_3_ (Nit) or normal drinking water. After 1 week, half of each group was fed with a choline-deficient high-fat diet (CDHFD), and the others were fed with a NCD for 16 weeks (see Fig. 1a). **n**, **o** Proportions of hepatic MoMFs (shown as CD11b^high^F4/80^int^ cell circles in **n**), KCs (shown as CD11b^int^F4/80^high^ cell circles in **n**), NKs, NKTs, CD4^+^ and CD8^+^ T cells of NCD and CDHFD groups were assessed via flow cytometry plus graphs (Kruskal‒Wallis test for MoMFs and NKs, one-way ANOVA with a post hoc test for KCs, NKTs, and CD4^+^ and CD8^+^ T cells, *P < 0.05, **P < 0.01, *n* = 5, biological replicates). **p** Absolute cell numbers of hepatic MoMFs, KCs, NKs, NKTs, CD4^+^ and CD8^+^ T cells of NCD and CDHFD groups were assessed via flow cytometry plus a graph (Kruskal‒Wallis test for KCs, NKs, CD4^+^ T cells, One-way ANOVA with a post hoc test for MoMFs, NKTs, and CD8^+^ T cells, *P < 0.05, **P < 0.01, *n* = 5, biological replicates). **q** Representative F4/80-stained mouse liver sections plus a graph showing F4/80-positive staining in NCD and CDHFD groups. Scale bars: 100 μm (one-way ANOVA with a post hoc test, *P < 0.05, *n* = 5, biological replicates). **r** Proportions of CD11C^+^ MoMFs and CD206^+^ MoMFs in the livers of the NCD and CDHFD groups (Kruskal‒Wallis test for CD206^+^ MoMFs, one-way ANOVA with a post hoc test for CD11C^+^ MoMFs, *P < 0.05, **P < 0.01, *n* = 5, biological replicates). **s** Absolute cell numbers of hepatic CD11C^+^ MoMFs and CD206^+^ MoMFs of the NCD and CDHFD groups (Kruskal‒Wallis test, *P < 0.05, **P < 0.01, *n* = 5, biological replicates). Relative hepatic *Inos, Il12b, Arg1*, and *Cd206* mRNA expression of the NCD and CDHFD groups (Kruskal‒Wallis test for *Inos, Il12b, and Arg1*, one-way ANOVA with a post hoc test for *Cd206*, *P < 0.05, **P < 0.01, *n* = 5, biological replicates). **t** Apoptosis (annexin V^+^ cells) of hepatic MoMFs of NCD and CDHFD groups (one-way ANOVA with a post hoc test, *P < 0.05, *n* = 5, biological replicates). **u** Proportion of hepatic TNF-α^+^ CD11C^+^ MoMFs of NCD and CDHFD groups (one-way ANOVA with a post hoc test, *P < 0.05, **P < 0.01, *n* = 5, biological replicates). **v**–**z** Another group of C57BL/6 mice received drinking water supplemented with 4 mM NaNO_3_ (Nit) or normal drinking water. After 1 week, half of each group was fed with a methionine/choline-deficient diet (MCD), and the other half was fed with a NCD for 4 weeks (see Supplementary Fig. 2c). **v** Proportions of hepatic MoMFs of the NCD and MCD groups (one-way ANOVA with a post hoc test, ***P < 0.001, *n* = 5, biological replicates). Absolute cell numbers of hepatic MoMFs of the NCD and MCD groups (one-way ANOVA with a post hoc test, **P < 0.01, ***P < 0.001, *n* = 5, biological replicates). **w** Proportions of hepatic CD11C^+^ MoMFs and CD206^+^ MoMFs of NCD and MCD groups (Kruskal‒Wallis test for CD206^+^ MoMFs, one-way ANOVA with a post hoc test for CD11C^+^ MoMFs, *P < 0.05, **P < 0.01, ***P < 0.001, *n* = 5, biological replicates). **x** Relative hepatic *Inos*, *Il12b*, *Arg1*, and *Cd206* mRNA expression of the NCD and MCD groups (Kruskal‒Wallis test for *Cd206*, one-way ANOVA with a post hoc test for *Inos, Il12b, and Arg1*, *P < 0.05, **P < 0.01, ***P < 0.001, *n* = 5, biological replicates). **y** Apoptosis (annexin V^+^ cells) of hepatic MoMFs of NCD and MCD groups (one-way ANOVA with a post hoc test, *P < 0.05, **P < 0.01, *n* = 5, biological replicates). **z** Proportion of hepatic TNF-α^+^ CD11C^+^ MoMFs of NCD and MCD groups (Kruskal‒Wallis test, *P < 0.05, **P < 0.01, *n* = 5, biological replicates)

To investigate whether NaNO_3_ attenuates metabolic abnormalities through a mechanism involving immunoregulatory processes, we detected the immune cell composition in mice. In these experiments, mice were induced with 12-week NCD or WD, followed by 12-week addition of 4 mM NaNO_3_ (Nit) in the drinking water. The animals were euthanized, after which plasma and liver samples were harvested (see Fig. 2c). The results revealed that the proportions and absolute cell numbers of bone marrow monocyte-derived macrophages (MoMFs), but not those of Kupffer cells (KCs), natural killer cells (NKs), NK T cells (NKTs), CD4^+^ and CD8^+^ T cells, in mouse livers were markedly decreased, as assessed via flow cytometry (Fig. 3a–c). A standard flow cytometric gating approach was employed to identify distinct immune cell populations within the liver (Supplementary Fig. 4a, b). Specifically, NaNO_3_ decreased the proportion and absolute cell number of CD11C^+^ (CD11C^+^CD206^−^) MoMFs (Fig. 3d, e), suggesting that NaNO_3_ has anti-inflammatory effects in the liver. NaNO_3_ increased the percentage of apoptotic MoMFs (Fig. 3f). The proportion of TNF-α-positive CD11C^+^ MoMFs was elevated in the livers of WD group, but was decreased by NaNO_3_ treatment (Fig. 3g). Additionally, with respect to KCs (defined in the liver as Tim4^+^Clec4F^+^^20^), flow cytometry analysis revealed that these cells did not exhibit any changes after nitrate treatment (Supplementary Fig. 4c). Interestingly, NaNO_3_ also reduced the WD-induced elevation in both the proportion and total absolute cell number of MoMFs, decreased the proportion of CD11C^+^ MoMFs and increased that of CD206^+^ (CD11C^−^CD206^+^) MoMFs in GAT in WD-fed mice (Fig. 3h–k). Moreover, NaNO_3_ increased the proportion of apoptotic MoMFs (Fig. 3l) and decreased that of TNF-α-positive CD11C^+^ MoMFs in the GAT of WD mice (Fig. 3m). Comparable outcomes were detected in the liver tissues of CDHFD group (Fig. 3n–u, Supplementary Fig. 4d–f) and MCD-fed mice (Fig. 3v–z, Supplementary Fig. 4g–j).

### NaNO_3_ directly regulates MoMFs polarization and function without affecting NO production in vitro

**Fig. 4:**
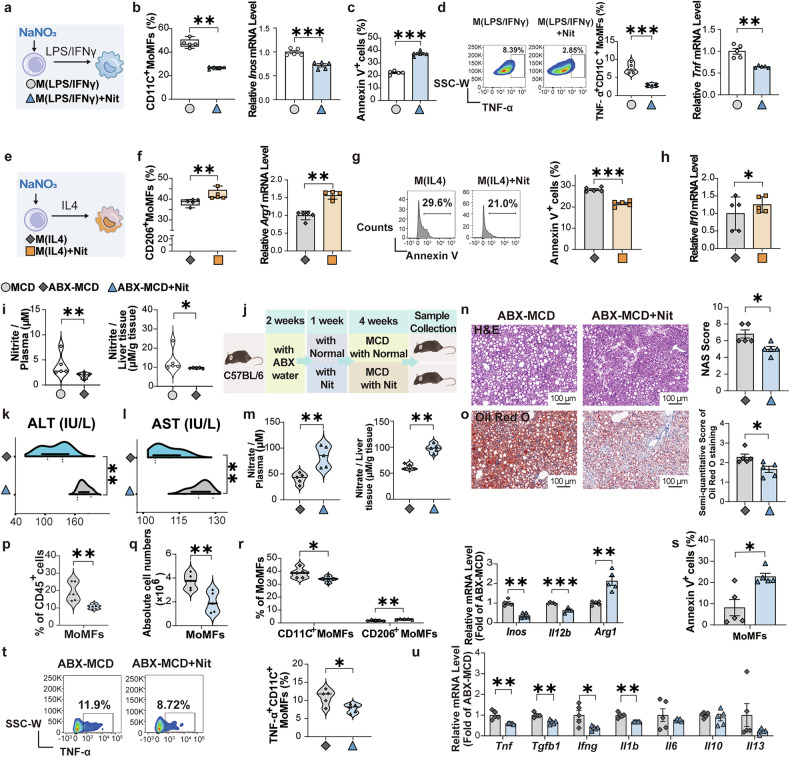
NaNO3 modulates MoMFs inflammatory responses and survival in vitro, and dietary NaNO3 inhibits metabolic disorders and liver inflammation in ABX mice. **a**–**h** Bone marrow-derived monocytes were harvested and treated with or without 2 mM NaNO_3_ for 48 h, induced in vitro to differentiate into MoMFs, and polarized into LPS/IFN-γ-stimulated MoMFs (M(LPS/IFNγ)) or IL-4-stimulated MoMFs (M(IL4)). **a** Schematic overview of the experiment with M(LPS/IFNγ). **b** Proportion of CD11C^+^ cells and relative *Inos* mRNA expression in M(LPS/IFNγ) (Kruskal‒Wallis test for CD11C^+^ cells, Student’s *t*-test for *Inos*, **P < 0.01, ***P < 0.001, *n* = 5, biological replicates). **c** Apoptosis (annexin V^+^ cells) in M(LPS/IFNγ) (Student’s *t*-test, ***P < 0.001, *n* = 5, biological replicates). **d** Proportion of TNF-α positive CD11C^+^ MoMFs plus graph and relative *Tnf* mRNA expression in M(LPS/IFNγ) (Student’s *t*-test, ***P < 0.001, *n* = 5, biological replicates). **e** Schematic overview of the experiment in M(IL4). **f** Proportion of CD206^+^ cells and relative *Arg1* mRNA expression in M(IL4) (Kruskal‒Wallis test for CD206^+^ cells, Student’s *t*-test for *Arg1*, **P < 0.01, *n* = 5, biological replicates). **g** Representative flow cytometric plots of apoptosis (annexin V^+^ cells) plus graph in M(IL4) (Student’s *t*-test, ***P < 0.001, *n* = 5, biological replicates). **h** Relative *Il10* mRNA expression in M(IL4) (Student’s *t*-test, *P < 0.05, *n* = 5, biological replicates). **i**–**u** C57BL/6 mice with broad-spectrum antibiotics (ABX) were generated. After 2 weeks, the mice received drinking water supplemented with 4 mM NaNO_3_ (Nit) or normal water for 1 week, after which all the mice were fed with a methionine/choline-deficient diet (MCD). A single oral ABX gavage was given to all the mice 2 weeks later. After another 2 weeks, the plasma and livers were collected. **i** Nitrite concentrations in the plasma and livers of MCD and MCD-ABX groups (Kruskal‒Wallis test, *P < 0.05, **P < 0.01, *n* = 5, biological replicates). **j** Schematic overview of the experiment in MCD-fed ABX mice treated with or without NaNO_3_. **k** Plasma alanine aminotransferase (ALT) levels in MCD-ABX groups treated with or without NaNO_3_ (Student’s *t*-test, **P < 0.01, *n* = 5, biological replicates). **l** Plasma aspartate aminotransferase (AST) levels in MCD-ABX groups treated with or without NaNO_3_ (Student’s *t*-test, **P < 0.01, *n* = 5, biological replicates). **m** Nitrate concentrations in the plasma and livers of MCD-ABX groups treated with or without NaNO_3_ (Kruskal‒Wallis test for liver, Student’s *t*-test for plasma, **P < 0.01, *n* = 5, biological replicates). **n**, **o** Representative H&E- and Oil Red O-stained liver sections plus a graph showing the NAS and semiquantitative score. Scale bars: 100 μm (Kruskal‒Wallis test, *P < 0.05, *n* = 5, biological replicates). **p** Proportions of MoMFs in the livers of MCD-ABX groups treated with or without NaNO_3_ (Kruskal‒Wallis test, **P < 0.01, *n* = 5, biological replicates). **q** Absolute cell numbers of hepatic MoMFs of MCD-ABX groups treated with or without NaNO_3_ (Student’s *t*-test, **P < 0.01, *n* = 5, biological replicates). **r** Proportions of hepatic CD11C^+^ MoMFs and CD206^+^ MoMFs and relative *Inos, Il12b*, and *Arg1* mRNA expression of MCD-ABX groups treated with or without NaNO_3_ (Student’s *t*-test for CD11C^+^ MoMFs, CD206^+^ MoMFs, *Il12b*, and *Arg1*, Kruskal–Wallis test for *Inos*, *P < 0.05, **P < 0.01, ***P < 0.001, *n* = 5, biological replicates). **s** Apoptosis (annexin V^+^ cells) of hepatic MoMFs of MCD-ABX groups treated with or without NaNO_3_ (Kruskal‒Wallis test, *P < 0.05, *n* = 5, biological replicates). **t** Proportion of hepatic TNF-α positive CD11C^+^ MoMFs of the MCD-ABX groups treated with or without NaNO_3_ (Student’s *t*-test, *P < 0.05, *n* = 5, biological replicates). **u** Relative mRNA expression of hepatic pro-inflammatory and anti-inflammatory cytokine factors of MCD-ABX groups treated with or without NaNO_3_ (Kruskal‒Wallis test for *Il13*, Student’s *t*-test for others, *P < 0.05, **P < 0.01, *n* = 5, biological replicates)

To investigate whether NaNO_3_ directly regulates MoMFs function, bone marrow cells were harvested and subsequently subjected to NaNO_3_. After 48 h, LPS/IFN-γ or IL-4 were used to induce CD11C^+^ or CD206^+^ MoMFs. After confirming the concentration (Supplementary Fig. 5a–f), we found that NaNO_3_ decreased the CD11C^+^ cell proportion and *Inos* mRNA expression and increased apoptosis in these cells (Fig. 4a–c). NaNO_3_ reduced TNF-α positive CD11C^+^ MoMFs, as determined through flow cytometric and quantitative real-time PCR analyses (Fig. 4d). The opposite effects were observed in MoMFs stimulated with IL-4: upregulation of the CD206^+^ cell proportion and *Arg1* mRNA expression and downregulation of apoptosis after NaNO_3_ treatment (Fig. 4e–g). NaNO_3_ increased *Il10* mRNA expression in CD206^+^ MoMFs (Fig. 4h). Nitrate can be metabolized into NO, and NO plays a crucial role in endothelial health. Endothelial cells also regulate metabolic function.^21^ We determined the protein expression level of eNOS in liver tissues via western blotting and found that its expression was not significantly altered by nitrate treatment under experimental conditions in mice (Supplementary Fig. 5g, h). In addition, we detected the intracellular nitrate concentration and found that it significantly increased after nitrate supplementation, while there were no marked alterations in total nitrosylation (SNO) or NO within the cells (Supplementary Fig. 5i–k).

### Blocking NO production from oral and enteral symbiotic bacteria fails to inhibit NaNO_3_-mediated regulation of MoMFs polarization and function in mice

Findings from the aforementioned in vitro studies indicate that NaNO_3_ may regulate MoMFs function without affecting NO production. To minimize the potential impact of NO production from oral and enteral symbiotic bacteria on our results in vivo, C57BL/6 mice were subsequently exposed to broad-spectrum antibiotics (ABX)^22^ and then subjected to MCD treatment. The findings showed that the plasma and liver nitrite levels were markedly lower in ABX-treated MCD-fed mice than in untreated MCD-fed mice (Fig. 4i). Evaluation of the results under conditions with or without NaNO_3_ supplementation (Fig. 4j) revealed decreased ALT and AST levels (Fig. 4k, l). After NaNO_3_ treatment, the plasma and liver nitrate levels were markedly increased in the mice treated with antibiotics than in the control mice (Fig. 4m). Histological analysis revealed that NaNO_3_ attenuated hepatic inflammation and fat accumulation (Fig. 4n, o). Flow cytometry analysis revealed that NaNO_3_ decreased the proportion and absolute cell number of MoMFs and the proportion of CD11C^+^ MoMFs and increased the proportion of CD206^+^ MoMFs in the livers (Fig. 4p–r). NaNO_3_ downregulated the levels of *Inos* and *Il12b* mRNAs and elevated the level of *Arg1* mRNA in the livers of ABX-exposed MCD-fed mice (Fig. 4r). NaNO_3_ increased the proportion of apoptotic MoMFs (Fig. 4s) and decreased that of TNF-α-positive CD11C^+^ MoMFs (Fig. 4t). In ABX-treated MCD-fed mice, the transcriptional activity of pro-inflammatory cytokine genes was also significantly downregulated after NaNO_3_ treatment (Fig. 4u).

### Sialin loss essentially abolishes NaNO_3_-mediated modulation of MoMFs function in vitro and in bone marrow-reconstituted mice

**Fig. 5:**
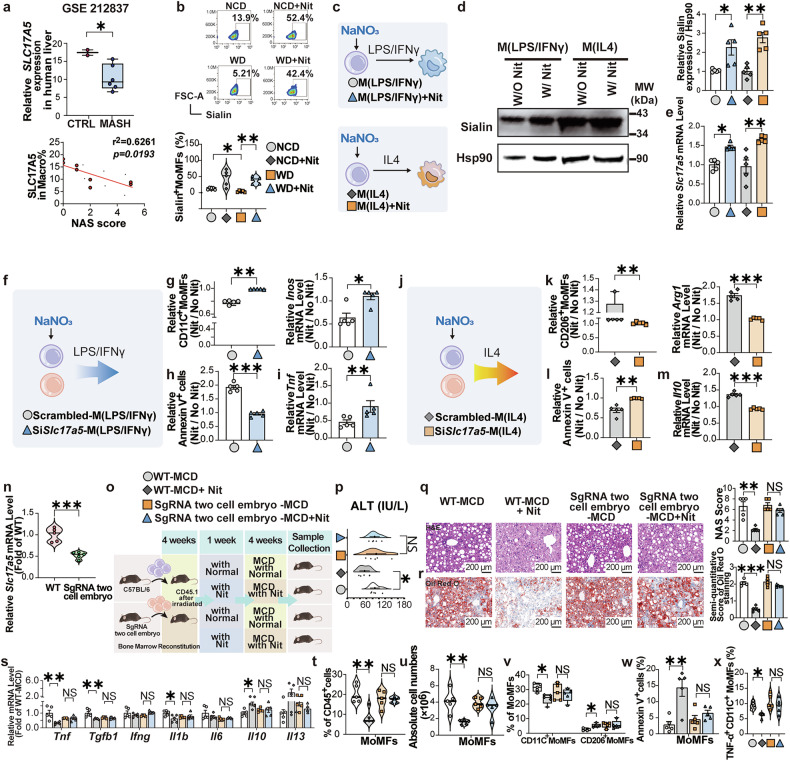
Sialin plays a key role in the modulation of MoMFs by NaNO_3_ in vitro and in vivo. **a** Analysis of single-cell sequencing data reveals sialin expression levels in human hepatic macrophages and correlation analysis with NAS score (Student’s *t*-test for sialin expression, nonparametric Spearman’s test for correlation analysis, *P < 0.05, CTRL *n* = 2, MASH *n* = 6, biological replicates). **b** Proportions of sialin-positive bone marrow monocyte-derived macrophages (MoMFs) plus graphs of normal diet (NCD)- and western diet (WD)-fed mice (one-way ANOVA with a post hoc test, *P < 0.05, **P < 0.01, *n* = 5, biological replicates). **c**–**e** Bone marrow-derived monocytes were harvested and treated with or without 2 mM NaNO_3_ for 48 h, induced in vitro to differentiate into MoMFs, and polarized into LPS/IFN-γ-stimulated MoMFs (M(LPS/IFNγ)) or IL-4-stimulated MoMFs (M(IL4)). **c** Schematic overview of the experiments with M(LPS/IFNγ) and M(IL4). **d** Relative expression of sialin (normalized to Hsp90) in M(LPS/IFNγ) and M(IL4) (one-way ANOVA with a post hoc test, *P < 0.05, **P < 0.01, *n* = 5, biological replicates). **e** Relative *Slc17a5* mRNA level in M(LPS/IFNγ) and M(IL4) (Kruskal‒Wallis test, *P < 0.05, **P < 0.01, *n* = 5, biological replicates). **f**–**m** Bone marrow-derived monocytes were harvested and treated with or without 2 mM NaNO_3_ for 48 h, transfected with *Slc17a5* or scrambled siRNA (control) in vitro, induced to differentiate into MoMFs, and polarized into M(LPS/IFNγ) or M(IL4). **f** Schematic overview of the experiments involving M(LPS/IFNγ) with *Slc17a5* or scrambled siRNA. **g** Proportion of CD11C^+^ cells and relative *Inos* mRNA level in M(LPS/IFNγ) treated with *Slc17a5* or scrambled siRNA (Kruskal‒Wallis test, *P < 0.05, **P < 0.01, *n* = 5, biological replicates). **h** Apoptosis (annexin V^+^ cells) in M(LPS/IFNγ) with *Slc17a5* or scrambled siRNA (Student’s *t*-test, ***P < 0.001, *n* = 5, biological replicates). **i** Relative *Tnf* mRNA expression in M(LPS/IFNγ) treated with *Slc17a5* or scrambled siRNA (Kruskal‒Wallis test, **P < 0.01, *n* = 5, biological replicates). **j** Schematic overview of the experiment involving M(IL4) with *Slc17a5* or scrambled siRNA. **k** Proportion of CD206^+^ cells and relative *Arg1* mRNA expression in M(IL4) treated with *Slc17a5* or scrambled siRNA (Kruskal‒Wallis test for CD206^+^ cells, Student’s *t*-test for *Arg1*, **P < 0.01,***P < 0.001, *n* = 5, biological replicates). **l** Apoptosis (annexin V^+^ cells) in M(IL4) treated with *Slc17a5* or scrambled siRNA (Student’s *t*-test, **P < 0.01, *n* = 5, biological replicates). **m** Relative *Il10* mRNA expression in M(IL4) treated with *Slc17a5* or scrambled siRNA (Student’s *t-*test, ***P < 0.001, *n* = 5, biological replicates). **n**–**x** Irradiated CD45.1 congenic mice were transplanted with donor bone marrow, wild-type (WT) C57BL/6 mice, or *Slc17a5* sgRNA two-cell embryo mice. After 4 weeks, half of each group received drinking water with 4 mM NaNO_3_ (Nit), while the other half had access to normal drinking water. After 1 week, all the mice were fed with a daily methionine/choline-deficient diet (MCD). After another 4 weeks, all the mice were sacrificed, and the plasma and liver were collected. **n** Relative *Slc17a5* mRNA expression in bone marrow from WT C57BL/6 mice and *Slc17a5* sgRNA two-cell embryo mice prior to bone marrow transplantation into irradiated CD45.1 congenic mice (Student’s *t*-test, ***P < 0.001, *n* = 5, biological replicates). **o** Schematic overview of the transplantation experiment. **p** Plasma alanine transaminase (ALT) levels in transplanted mice (one-way ANOVA with a post hoc test, *P < 0.05, NS no significance, *n* = 5, biological replicates). **q**, **r** Representative H&E-stained liver sections plus a graph showing the MASLD activity score (NAS) and representative Oil Red O-stained liver sections plus a graph showing the semiquantitative score. Scale bars: 200 μm (Kruskal‒Wallis test for NAS, one-way ANOVA with a post hoc test for Oil Red O staining, **P < 0.01, ***P < 0.001, NS no significance, *n* = 5, biological replicates). **s** Relative mRNA level of hepatic pro-inflammatory and anti-inflammatory cytokine factors of transplanted mice (one-way ANOVA with a post hoc test, *P < 0.05, **P < 0.01, NS no significance, *n* = 5, biological replicates). **t** Proportion of MoMFs in the livers of transplanted mice (Kruskal‒Wallis test, **P < 0.01, NS no significance, *n* = 5, biological replicates). **u** Absolute cell numbers of MoMFs in the livers of transplanted mice (one-way ANOVA with a post hoc test, **P < 0.01, NS no significance, *n* = 5, biological replicates). **v** Proportions of CD11C^+^ MoMFs, and CD206^+^ MoMFs in the livers of transplanted mice (Kruskal‒Wallis test for CD206^+^ MoMFs, one-way ANOVA with a post hoc test for CD11C^+^ MoMFs, *P < 0.05, NS no significance, *n* = 5, biological replicates). **w** Apoptosis (annexin V^+^ cells) of MoMFs, CD11C^+^ MoMFs, and CD206^+^ MoMFs in the livers of transplanted mice (one-way ANOVA with a post hoc test, **P < 0.01, NS no significance, *n* = 5, biological replicates). **x** Proportion of TNF-α positive CD11C^+^ MoMFs in the livers of transplanted mice (Kruskal‒Wallis test, *P < 0.05, NS no significance, *n* = 5, biological replicates)

Earlier research from our group established that sialin functions an essential role in nitrate transport. Evaluation of single-cell sequencing data from human databases^23^ revealed a high expression level of sialin in human macrophages, which was reduced in macrophages from MASH (previously NASH) patients (Fig. 5a). Furthermore, sialin expression exhibited an inverse correlation with NAS scores. The proportion of hepatic sialin-positive MoMFs was markedly lower in the WD group than in the NCD group and were restored by NaNO_3_ (Fig. 5b).

The findings from in vitro studies revealed that NaNO_3_ upregulated the levels of sialin in both protein and mRNA in CD11C^+^ or CD206^+^ MoMFs (Fig. 5c–e). siRNA-mediated knockdown of sialin expression largely decreased the effects of NaNO_3_ on the survival, proportion, and apoptosis of CD11C^+^ or CD206^+^ MoMFs (Supplementary Fig. 6a–c and Fig. 5f–m). NaNO_3_ did not markedly alter *Tnf* or *Il10* mRNA expression in CD11C^+^ or CD206^+^ MoMFs in the presence of sialin knockdown (Fig. 5i, m). Collectively, these findings indicated that sialin significantly contributed to the regulation of MoMFs by NaNO_3_ in vitro.

As a null/null mutation in *Slc17a5*, which encodes sialin, leads to early postnatal death,^24^ we employed mice with a heritable *Slc17a5* mutation, which we generated via CRISPR-Cas9 and microinjection of *Slc17a5* single-guide RNA (sgRNA) into one cell from two-cell stage murine embryos, as we previously described.^25^ To investigate whether sialin mediates NaNO_3_ modulation of MoMFs in vivo, we adoptively transferred bone marrow cells from *Slc17a5* sgRNA two-cell embryo mice or wild-type C57BL/6 mice to irradiated CD45.1 congenic recipient mice; the level of the mutated *Slc17a5* gene was detected in the bone marrow cells from the mutant mice (Fig. 5n). Four weeks after bone marrow reconstitution, the recipient mice received NaNO_3_ supplement or normal drinking water, and 1 week later, they were fed with a MCD or NCD for another 4 weeks (Fig. 5o). The results revealed that NaNO_3_ treatment significantly decreased plasma ALT levels, liver tissue inflammation and lipid accumulation in irradiated mice that had been transplanted with wild-type bone marrow but not in those that had received bone marrow from *Slc17a5* sgRNA two-cell embryo mice (Fig. 5p–r). In the *Slc17a5* sgRNA two-cell embryo bone marrow-reconstituted mice, NaNO_3_ did not cause marked alterations in hepatic inflammatory factors (Fig. 5s), proportions, absolute cell numbers, or apoptosis of MoMFs or their subsets (Fig. 5t–w), or TNF-α-positive CD11C^+^ MoMFs (Fig. 5x). Thus, we established that sialin is indispensable for the in vivo regulation of MoMFs by NaNO_3_.

### Sialin regulates macrophages polarization and function through the CtsL‒Nrf2 axis

**Fig. 6:**
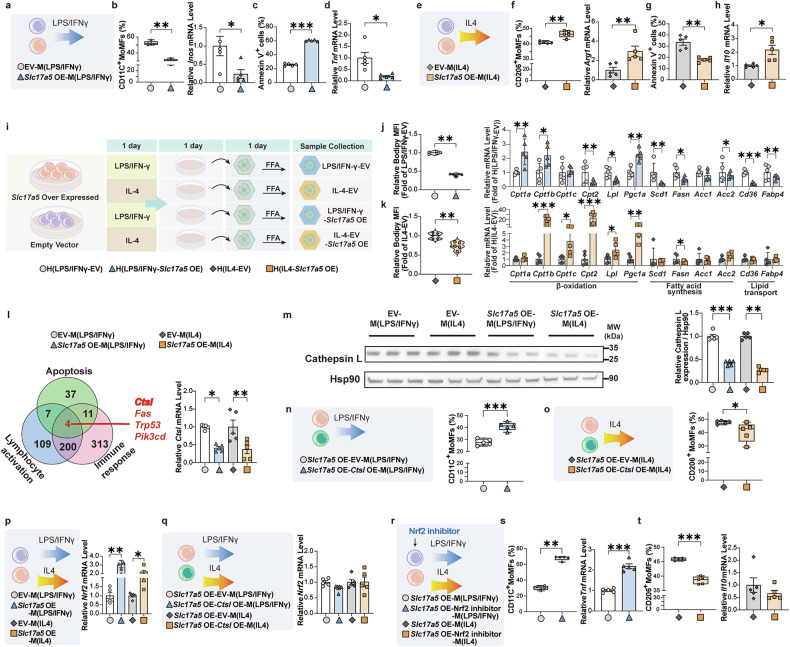
Sialin directly regulates macrophage polarization and function by downregulating CtsL expression and activating Nrf2. **a**–**h** Overexpression of sialin (*Slc17a5* OE) or empty vector (EV)-macrophages were cultured in vitro and polarized into LPS/IFN-γ-stimulated MoMFs (M(LPS/IFNγ)) or IL-4-stimulated MoMFs (M(IL4)). **a** Schematic overview of the experiments involving EV- and *Slc17a5* OE-M(LPS/IFNγ). **b** Proportion of CD11C^+^ cells and relative *Inos* mRNA expression in EV- and *Slc17a5* OE-M(LPS/IFNγ) (Kruskal‒Wallis test, *P < 0.05, **P < 0.01, *n* = 5, biological replicates). **c** Apoptosis (annexin V^+^ cells) in EV- and *Slc17a5* OE-M(LPS/IFNγ) (Student’s *t*-test, ***P < 0.001, *n* = 5, biological replicates). **d** Relative *Tnf* mRNA expression in EV- and *Slc17a5* OE-M(LPS/IFNγ) (Student’s *t*-test, *P < 0.05, *n* = 5, biological replicates). **e** Schematic overview of the experiments involving EV- and *Slc17a5* OE-M(IL4). **f** Proportion of CD206^+^ cells and relative *Arg1* mRNA expression in EV- and *Slc17a5* OE-M(IL4) (Kruskal‒Wallis test for *Arg1*, one-way ANOVA with a post hoc test for CD206^+^ cells, **P < 0.01, *n* = 5, biological replicates). **g** Apoptosis (annexin V^+^ cells) in EV- and *Slc17a5* OE-M(IL4) (one-way ANOVA with a post hoc test, **P < 0.01, *n* = 5, biological replicates). **h** Relative *Il10* mRNA expression in EV- and *Slc17a5* OE-M(IL4) (Student’s *t*-test, *P < 0.05, *n* = 5, biological replicates). **i**–**k** EV- or *Slc17a5* OE-macrophages were cultured in vitro and polarized into M(LPS/IFNγ) or M(IL4) for one day. Then, the culture medium was transferred to hepatocytes (H(LPS/IFNγ), H(IL4)), and free fatty acid (FFA) was added for one day. **i** Schematic overview of the experiments involving FFA-stimulated H(LPS/IFNγ) and H(IL4). **j** Relative MFI of Bodipy and mRNA expression in H(LPS/IFNγ) (Kruskal‒Wallis test for Bodipy, Student’s *t*-test for mRNA, *P < 0.05, **P < 0.01, ***P < 0.001, *n* = 5, biological replicates). **k** Relative MFI of Bodipy and mRNA expression in H(IL4) (Kruskal‒Wallis test for *Cpt1a, Pgc1a, Scd1, Acc1*, and *Cd36*, Student’s *t*-test for Bodipy and others, *P < 0.05, **P < 0.01, ***P < 0.001, *n* = 5, biological replicates). **l** mRNA alterations associated with the EV- or *Slc17a5* OE-macrophages were identified by RNA sequencing. Relative *Ctsl* mRNA expression (one-way ANOVA with a post hoc test, *P < 0.05, **P < 0.01, *n* = 5, biological replicates). **m** Relative protein expression of cathepsin L (normalized to Hsp90 expression) (Kruskal‒Wallis test for M(LPS/IFNγ), one-way ANOVA with a post hoc test for M(IL4), **P < 0.01, ***P < 0.001, *n* = 5, biological replicates). **n**, **o** *Slc17a5* OE-macrophages transfected with the *Ctsl* OE plasmid or EV in vitro were induced to differentiate into MoMFs and polarized into M(LPS/IFNγ) or M(IL4). **n** Proportion of CD11C^+^ cells in *Slc17a5* OE- and *Ctsl- Slc17a5* OE-M (LPS/IFNγ) (Student’s *t*-test, ***P < 0.001, *n* = 5, biological replicates). **o** Proportion of CD206^+^ cells in *Slc17a5* OE- and *Ctsl- Slc17a5* OE-M(IL4) populations (Student’s *t*-test, *P < 0.05, *n* = 5, biological replicates). **p** EV- or *Slc17a5* OE-macrophages were cultured in vitro and polarized into M(LPS/IFNγ) or M(IL4). Relative *Nrf2* mRNA expression in CD11C^+^ and CD206^+^ MoMFs (Kruskal‒Wallis test, *P < 0.05, **P < 0.01, *n* = 5, biological replicates). **q** EV- or *Slc17a5* OE-macrophages transfected with EV- or *Ctsl* OE plasmid in vitro were induced to differentiate into MoMFs and polarized into M(LPS/IFNγ) or M(IL4). Relative *Nrf2* mRNA expression in CD11C^+^ and CD206^+^ MoMFs (Kruskal‒Wallis test, *n* = 5, biological replicates). **r**–**t** EV- or *Slc17a5* OE-macrophages, treated with or without a Nrf2 inhibitor (ML385) in vitro, induced to differentiate into MoMFs, polarized into M(LPS/IFNγ) or M(IL4). **r** Schematic overview of the experiments involving ML385-induced M(LPS/IFNγ) and M(IL4). **s** Proportion of CD11C^+^ cells and relative *Tnf* mRNA expression in ML385-induced M(LPS/IFNγ) (Kruskal‒Wallis test for CD11C^+^ cells, Student’s *t*-test for *Tnf*, **P < 0.01, ***P < 0.001, *n* = 5, biological replicates). **t** Proportion of CD206^+^ cells and relative *Il10* mRNA expression in ML385-induced M(IL4) (Kruskal‒Wallis test for *Il10*, Student’s *t*-test for CD206^+^ cells, ***P < 0.001, *n* = 5, biological replicates)

To further verify whether sialin can regulate macrophage polarization, we generated macrophages with sialin overexpression (OE). We found that sialin OE reduced the proportion of CD11C^+^ MoMFs and the expression level of *Inos* mRNA (Supplementary Fig. 6d–f, Fig. 6a, b), increased the apoptosis of CD11C^+^ MoMFs (Fig. 6c), and decreased *Tnf* mRNA expression (Fig. 6d) upon LPS/IFN-γ stimulation. In contrast, sialin OE increased the proportion of CD206^+^ MoMFs and *Arg1* mRNA expression (Fig. 6e, f), decreased the apoptosis of CD206^+^ MoMFs (Fig. 6g), and increased *Il10* mRNA expression (Fig. 6h) upon IL-4 stimulation. To verify whether the intracellular nitrate concentration, SNO and NO levels were altered, we found that neither sialin OE, sialin knockdown, nor sialin knockdown followed by nitrate supplementation induced any significant changes (Supplementary Fig. 6g–i).

To determine whether sialin-overexpressing macrophages can induce lipid accumulation in hepatocytes, we cultured sialin OE or EV (empty vector) macrophages for 24 h and collected the culture media. We stimulated free fatty acid (FFA)-treated AML12 cells with media (Fig. 6i). As shown in Fig. 6j, k, sialin OE macrophage culture media decreased lipid accumulation in AML12 cells. Moreover, conditioned medium from sialin-overexpressing cells upregulated the transcriptional activity of fatty acid β-oxidation-related genes while suppressing fatty acid synthesis and lipid transport-associated genes in hepatocytes.

We next determined gene expression in LPS/IFN-γ-stimulated (CD11C^+^) or IL-4-stimulated (CD206^+^) macrophages (RAW264.7) with or without sialin OE via transcriptome sequencing. The results revealed that 4799 genes were regulated by sialin in both CD11C^+^ and CD206^+^ macrophages (Supplementary Fig. 6j, k). Gene ontology (GO) pathway analysis showed that these genes participate within leukocyte activation, apoptosis, myeloid cell differentiation and the innate immune response (Supplementary Fig. 6l). Further analysis revealed that *Ctsl* was involved in sialin regulation of apoptosis, leukocyte activation and the immune response of macrophages (Fig. 6l). Cathepsin L (CtsL, encoded by *Ctsl*) is a lysosomal and cytoplasmic protease that is participated in diverse cellular processes, especially as a potential marker for liver fibrosis.^26^ Expression of both the *Ctsl* gene and its corresponding protein CtsL was validated in CD11C^+^ and CD206^+^ macrophages with or without sialin OE. Moreover, their expression levels were markedly reduced in macrophages overexpressing sialin compared to those transduced with the empty vector (Fig. 6l, m), whereas other genes were not significantly altered (Supplementary Fig. 6m–o).

To more comprehensively elucidate the mechanistic basis of sialin-mediated regulation in MoMFs, we induced CtsL OE with and without sialin OE in MoMFs (Supplementary Fig. 6p, q). We found that in the presence of sialin OE, CtsL OE increased the proportion of LPS/IFN-γ-induced CD11C^+^ macrophages and decreased the proportion of IL-4-induced CD206^+^ macrophages (Fig. 6n, o). These in vitro experiments demonstrated that CtsL may play the main role in the sialin-mediated modulation of the inflammatory response and polarization of MoMFs.

Because the nuclear factor erythroid 2-related factor 2 (Nrf2) pathway is located downstream of CtsL,^27^ which reportedly modulates the inflammatory response of MoMFs,^28,29^ we investigated the expression of this transcription factor in our in vitro models. We found that sialin upregulated the mRNA expression of *Nrf2* in CD11C^+^ or CD206^+^ MoMFs (Fig. 6p). CtsL OE reversed the alteration in Nrf2 expression induced by sialin OE (Fig. 6q). Moreover, ML385, an Nrf2 inhibitor, significantly impaired the effects of sialin on CD11C^+^ or CD206^+^ MoMFs (Fig. 6r–t). *Ctsl* siRNA knockdown (Supplementary Fig. 6r, s) decreased the proportion of CD11C^+^ cells (Supplementary Fig. 6t) and *Inos* mRNA expression (Supplementary Fig. 6u) and upregulated the CD206^+^ cell proportion (Supplementary Fig. 6v) and *Arg1* mRNA expression (Supplementary Fig. 6w), whereas *Ctsl* knockdown upregulated *Nrf2* mRNA expression (Supplementary Fig. 6x).

To gain deeper insight into sialin’s function in mediating the effects of NaNO₃ within MoMFs, we performed RNA sequencing of NaNO_3_-treated CD11C^+^ or CD206^+^ MoMFs with and without sialin knockdown (Supplementary Fig. 7a). After NaNO_3_ treatment, the expression of 1657 genes changed in CD11C^+^ MoMFs, and of these genes, 812 genes were regulated by sialin (Supplementary Fig. 7b). In CD206^+^ MoMFs, the expression of 2965 genes was altered after NaNO_3_ treatment, of which 1409 genes were regulated by sialin (Supplementary Fig. 7b). We found that 242 genes were regulated by sialin in both CD11C^+^ and CD206^+^ MoMFs after NaNO_3_ treatment. GO and KEGG pathway analyses revealed that these 242 genes are also involved in cell activation, apoptosis, the immune response, and leukocyte differentiation (Supplementary Fig. 7c, d). Among the 242 genes screened, *Ctsl* was also a common differentially expressed gene at the intersection of apoptosis, the immune response, and lymphocyte activation (Supplementary Fig. 8a). We therefore examined its mRNA and protein expression levels in NaNO_3_-treated MoMFs exposed to LPS/IFN-γ or IL-4 for inducing CD11C^+^ or CD206^+^ macrophage polarization, finding that mRNA and protein levels were markedly downregulated in those of NaNO_3_-untreated MoMFs (Supplementary Fig. 8b, c). To provide additional evidence for sialin’s regulatory function in controlling CtsL expression, we transfected CD11C^+^ or CD206^+^ MoMFs with *Slc17a5* or scrambled siRNA after treatment with or without NaNO_3_. As shown in Supplementary Fig. 8d, e, sialin knockdown significantly increased *Ctsl* mRNA and protein levels in NaNO_3_-treated MoMFs compared with those in untreated cells. We overexpressed CtsL in primary MoMFs from C57BL/6 mice and treated them with or without NaNO_3._ Subsequently, the cells were stimulated to differentiate using either LPS/IFN-γ or IL-4. The experimental outcomes revealed that CtsL OE impaired the inhibitory effect of NaNO_3_ on LPS/IFN-γ-stimulated MoMFs, as indicated by significant increases in the CD11C^+^ cell proportion (Supplementary Fig. 8f) and *Inos* mRNA expression (Supplementary Fig. 8g). CtsL OE reduced the enhancing effect of NaNO_3_ on IL-4-stimulated MoMFs, as indicated by significant decreases in the CD206^+^ cell proportion (Supplementary Fig. 8h) and *Arg1* mRNA expression (Supplementary Fig. 8i). Nrf2 upregulation was observed after NaNO_3_ stimulation (Supplementary Fig. 8j). ML385 weakened the regulatory effects of NaNO_3_ on CD11C^+^ and CD206^+^ MoMFs (Supplementary Fig. 8k, l). Next, we downregulated *Slc17a5* expression and observed that in the presence of NaNO_3_, the level of *Nrf2* was markedly reduced in the *Slc17a5* siRNA-treated cells compared to the scrambled siRNA group (Supplementary Fig. 8m). Furthermore, CtsL OE downregulated the NaNO_3_-induced increase in *Nrf2* expression in MoMFs (Supplementary Fig. 8n).

### Sialin blocks Rel nuclear translocation to inhibit CtsL expression and activate the Nrf2 pathway

**Fig. 7:**
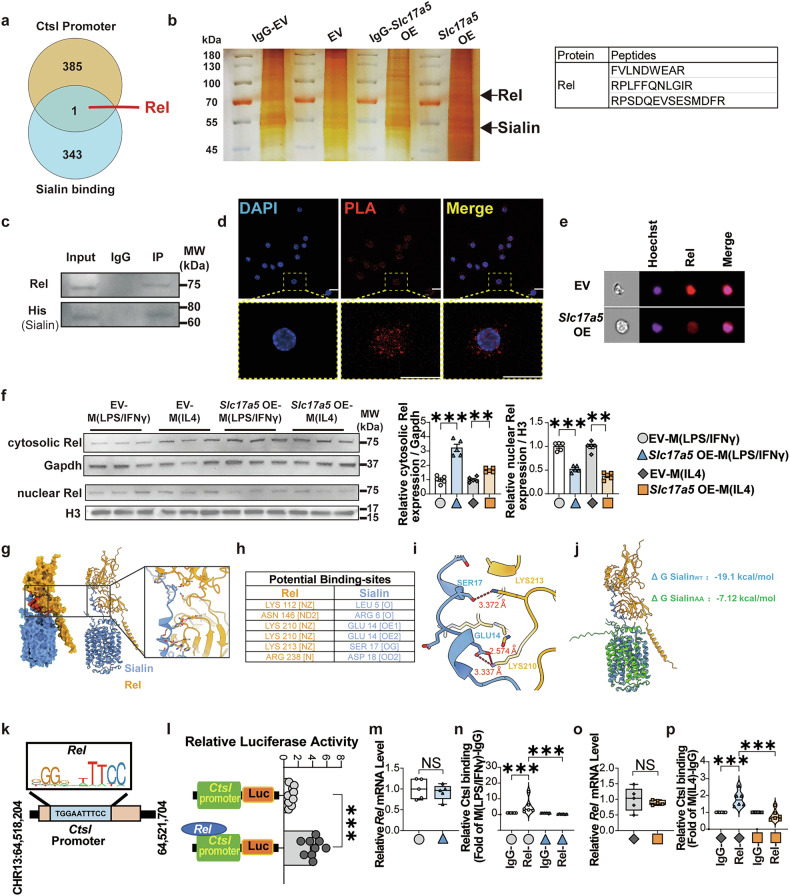
Sialin activates the Nrf2 pathway by inhibiting the nuclear translocation of Rel and downregulating CtsL expression. **a** Overexpression of sialin (*Slc17a5* OE)- or empty vector (EV) -macrophages were cultured in vitro, and polarized into LPS/IFN-γ-stimulated MoMFs (M(LPS/IFNγ)) or IL-4-stimulated MoMFs (M(IL4)). Rel in both the IP-MS and *Ctsl* promoter. **b** IP‒MS and analysis of the IgG-EV, EV, IgG-*Slc17a5* OE, and *Slc17a5* OE groups. Rel was screened. **c** CoIP of Rel binding to sialin. **d** Endogenous interaction between Rel and sialin in macrophages via proximity ligation assay (PLA). **e** Rel in the nucleus and cytoplasm of EV- or *Slc17a5* OE-macrophages. **f** Relative protein cathepsin L level within the nucleus and cytoplasm of EV- and *Slc17a5* OE- macrophages (Kruskal‒Wallis test for M(IL4), one-way ANOVA with a post hoc test for M(LPS/IFNγ), **P < 0.01, ***P < 0.001, *n* = 5, biological replicates). **g** Overall view of the docking model generated via the Z-DOCK server. Sialin is shown in blue, Rel in orange, and the complex surface in red. The dashed box indicates the predicted interaction interface, which is located within the flexible, hydrophilic N-terminal cytoplasmic region of sialin. **h** Predicted binding sites between the sialin–Rel complex via PDBePISA tools. **i** Close-up view of representative hydrogen bond interactions: SER17–LYS210 (2.574 Å), GLU14–LYS213 (3.337 Å), and a third pair at 3.372 Å. **j** Molecular docking of wild-type (WT) sialin and the alanine-substituted mutant (sialin-AA, SER17/GLU14→Ala). The mutant shows a higher binding free energy (WT: ΔG = −19.1 kcal/mol, AA: ΔG = −7.12 kcal/mol). **k** The TFBSs of Rel binding sites from the transcription start site of *Ctsl* were predicted. **l** Relative luciferase activity of Rel-Ctsl binding (Student’s *t*-test, ***P < 0.001, *n* = 9, biological replicates). **m** Relative *Rel* expression of EV- or *Slc17a5* OE-macrophages polarized into M(LPS/IFNγ) (Student’s *t*-test, NS no significance, *n* = 5, biological replicates). **n** Fold change in Rel binding to the *Ctsl* promoter (Kruskal‒Wallis test, ***P < 0.001, *n* = 6, biological replicates). **o** Relative *Rel* expression of EV- or *Slc17a5* OE-macrophages polarized into M(IL4) (Student’s *t*-test, NS no significance, *n* = 5, biological replicates). **p** Fold change in Rel binding to the *Ctsl* promoter (one-way ANOVA with a post hoc test, ***P < 0.001, *n* = 6, biological replicates)

As an initial step to investigate how sialin regulates CtsL expression, we employed protein analysis via immunoprecipitation and mass spectrometry (IP-MS). We screened potential sialin-binding proteins in sialin OE cells and detected high expression of Rel (Fig. 7a–c). Rel (commonly referred to as c-Rel and encoded by the *Rel* gene) belongs to the nuclear factor κB (NF-κB) pathway.^30^ Rel signaling of macrophages is profibrogenic and regulates plasticity.^31^ Rel is known to engage with multiple protein classes beyond those belonging to NF-κB families.^32–35^ The proximity ligation assay (PLA) revealed a strong endogenous interaction between Rel and sialin in macrophages (Fig. 7d). Imaging cytometry and western blot analysis revealed that sialin OE enhanced the cytosolic accumulation of Rel while reducing its nuclear localization, suggesting that sialin may inhibit Rel translocation into the nucleus (Fig. 7e, f).

To investigate the possible binding interface between sialin and Rel, we conducted rigid-body molecular docking. As shown in Fig. 7g, the overall docking model (sialin in blue, Rel in orange; surface colored red) reveals a well-defined interaction interface (highlighted by a dashed box). Sialin exhibits a classical major facilitator superfamily (MFS) architecture, characterized by 12 transmembrane helices (TMs) that form two pseudo-symmetric regions: an N-terminal domain comprising TM1 to TM6 and a C-terminal domain consisting of TM7 to TM12. Notably, the molecular docking results indicate that the interaction interface with Rel is located within the N-terminal cytoplasmic region outside the membrane-spanning segments. This flexible, hydrophilic region contains multiple polar and charged residues, making it well-suited for protein–protein interactions. Structural analysis of this interface revealed a dense network of hydrogen bonds, suggesting strong binding affinity. Combining analysis by docking and manual structural inspection identified several key contact residues at the interface (Fig. 7h). Among these, three representative hydrogen bond interactions were observed: SER17–LYS210 (2.574 Å), GLU14–LYS213 (3.337 Å), and a third contact at 3.372 Å (Fig. 7i). These distances fall within the typical hydrogen bonding range and support a stable interaction interface. To evaluate the functional relevance of these interactions, we performed in silico mutagenesis of SER17 and GLU14 to alanine, generating a double mutant construct termed sialin-AA. Molecular docking of the AA mutant with Rel (Fig. 7j) revealed a significant increase in binding free energy (WT: ΔG = −19.1 kcal/mol; AA mutant: ΔG = −7.12 kcal/mol), accompanied by marked conformational changes in sialin. These observations support the critical role of SER17 and GLU14 in mediating the sialin–Rel interaction and validate the reliability of the predicted interface.

A potential Rel transcription factor-binding site in the promoter of *Ctsl* was identified (Fig. 7k). A dual-luciferase reporter assay revealed that Rel transcriptionally regulated *Ctsl* expression (Fig. 7l). CUT&Tag analysis revealed that Rel binding to the *Ctsl* promoter was significantly decreased by sialin OE in CD11C^+^ or CD206^+^ MoMFs; interestingly, sialin OE did not affect *Rel* mRNA expression in cells (Fig. 7m–p). Collectively, these reveal that Rel serves a critical role in the mediation of *Ctsl* expression by sialin.

Validation of key signals was conducted in liver tissue through fluorescent multiplex immunohistochemistry (mIHC) analyses. The number of CtsL^+^ MoMFs was greater in the WD-induced model and lower after NaNO_3_ supplementation. Moreover, the level of Nrf2 in the hepatic monocytes of the CDHFD-induced model decreased, whereas it increased after NaNO_3_ administration (Supplementary Fig. 8o–q).

### NaNO_3_ modulates inflammatory responses and survival in human macrophages

**Fig. 8:**
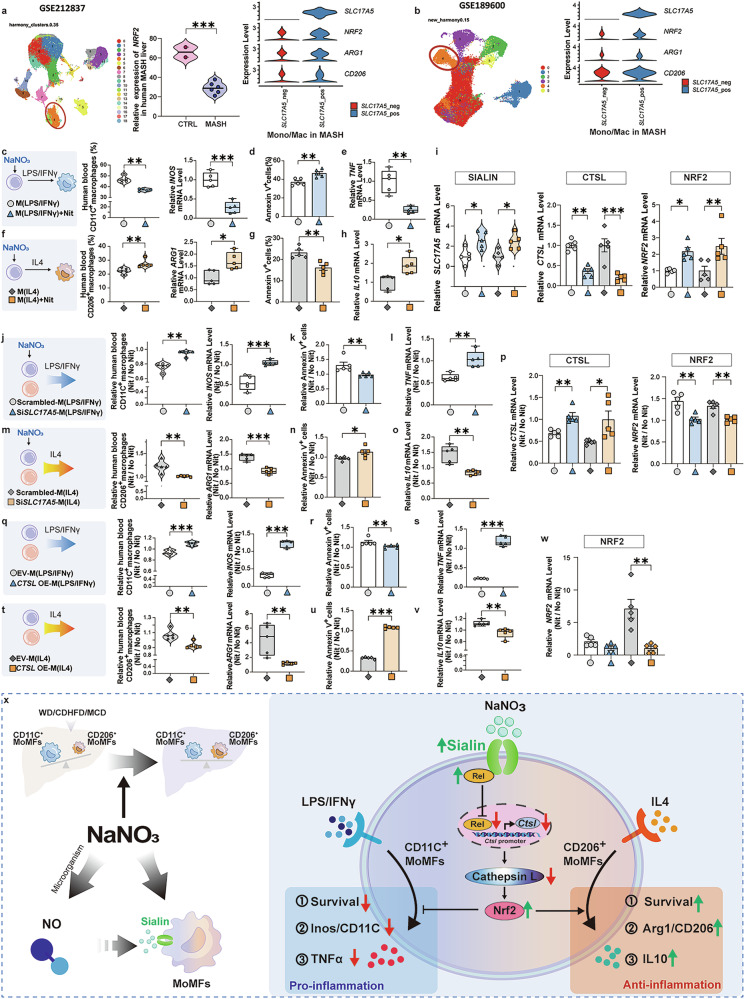
NaNO_3_ regulates the inflammatory response and survival of human macrophages in vitro. **a** Analysis of single-cell sequencing data from the GSE212837 dataset revealed *NRF2* expression in macrophages and *NRF2, ARG1*, and *CD206* expression in *SLC17A5-*positive and *SLC17A5-*negative macrophages (Student’s *t*-test, ***P < 0.001, CTRL *n* = 2, MASH *n* = 6, biological replicates). **b** Evaluation of single-cell sequencing data from the GSE189600 dataset revealed *NRF2, ARG1*, and *CD206* expression in *SLC17A5-*positive and *SLC17A5*-negative macrophages. **c-i** Human blood monocytes were cultured in vitro, treated with or without 2 mM NaNO_3_, induced to differentiate into macrophages, and polarized into LPS/IFN-γ-stimulated MoMFs (M(LPS/IFNγ)) or IL-4-stimulated MoMFs (M(IL4)). **c** Schematic overview of experiment in M(LPS/IFNγ). Proportion of CD11C^+^ cells and relative *INOS* expression (Student’s *t*-test, **P < 0.01, ***P < 0.001, *n* = 5, biological replicates). **d** Apoptosis (annexin V^+^ cells) in M(LPS/IFNγ) (Student’s *t*-test, **P < 0.01, *n* = 5, biological replicates). **e** Relative *TNF* mRNA level in M(LPS/IFNγ) (Student’s *t*-test, **P < 0.01, *n* = 5, biological replicates). **f** Schematic overview of the experiment in M(IL4). Proportion of CD206^+^ cells and relative *ARG1* mRNA expression (Kruskal–Wallis test for CD206^+^ cells, Student’s *t*-test for *ARG1*, *P < 0.05, **P < 0.01, *n* = 5, biological replicates). **g** Apoptosis (annexin V^+^ cells) in M(IL4) (Student’s *t*-test, **P < 0.01, *n* = 5, biological replicates). **h** Relative *IL10* mRNA level in M(IL4) (Student’s *t*-test, *P < 0.05, *n* = 5, biological replicates). **i** Relative *SLC17A5*, *CTSL* and *NRF2* mRNA level in M(LPS/IFNγ) and M(IL4) (One-way ANOVA with a post hoc test for *SLC17A5* and *CTSL*, Kruskal–Wallis test for *NRF2*, *P < 0.05, **P < 0.01, ***P < 0.001, *n* = 5, biological replicates). **j**–**p** Human blood monocytes were treated with or without 2 mM NaNO_3_ and then transfected with *SLC17A5* siRNA or scrambled siRNA in vitro, and then polarized into M(LPS/IFNγ) or M(IL4). **j** Schematic overview of the experiments involving *SLC17A5* or scrambled siRNA of M(LPS/IFNγ). Proportion of CD11C^+^ cells and relative *INOS* expression (Student’s *t*-test, **P < 0.01, ***P < 0.001, *n* = 5, biological replicates). **k** Apoptosis (annexin V^+^ cells) in *SLC17A5* or scrambled siRNA-treated M(LPS/IFNγ) (Student’s *t*-test, **P < 0.01, *n* = 5, biological replicates). **l** Relative *TNF* mRNA expression in *SLC17A5* or scrambled siRNA-treated M(LPS/IFNγ) (Kruskal‒Wallis test, **P < 0.01, *n* = 5, biological replicates). **m** Schematic overview of the experiments involving *SLC17A5* or scrambled siRNA-treated M(IL4). Proportion of CD206^+^ cells and relative *ARG1* mRNA expression (Student’s *t*-test, **P < 0.01, ***P < 0.001, *n* = 5, biological replicates). **n** Apoptosis (annexin V^+^ cells) in *SLC17A5* or scrambled siRNA of M(IL4) (Student’s *t*-test, *P < 0.05, *n* = 5, biological replicates). **o** Relative *IL10* mRNA expression in *SLC17A5* or scrambled siRNA-transfected M(IL4) (Student’s *t*-test, **P < 0.01, *n* = 5, biological replicates). **p** Relative *CTSL* and *NRF2* mRNA expression in *SLC17A5* or scrambled siRNA of M(LPS/IFNγ) and M(IL4) (Kruskal‒Wallis test for M(LPS/IFNγ), Student’s *t*-test for M(IL4), *P < 0.05, **P < 0.01, *n* = 5, biological replicates). **q**–**w** Human blood monocytes were treated with or without 2 mM NaNO_3_, transfected with a *CTSL* overexpression plasmid (OE) or an empty vector (EV) in vitro, and polarized into M(LPS/IFNγ) or M(IL4). **q** Schematic overview of the experiments involving EV- or *CTSL* OE-M(LPS/IFNγ). Proportion of CD11C^+^ cells and relative *INOS* expression (Student’s *t*-test, ***P < 0.001, *n* = 5, biological replicates). **r** Apoptosis (annexin V^+^ cells) in EV- or *CTSL* OE-M(LPS/IFNγ) (Kruskal‒Wallis test, **P < 0.01, *n* = 5, biological replicates). **s** Relative *TNF* mRNA expression in EV- or *CTSL* OE-M(LPS/IFNγ) (Student’s *t*-test, ***P < 0.001, *n* = 5, biological replicates). **t** Schematic overview of the experiments on the EV- or *CTSL* OE-M(IL4). Proportion of CD206^+^ cells and relative *ARG1* mRNA expression (Kruskal‒Wallis test for CD206^+^ cells, Student’s *t*-test for *ARG1*, **P < 0.01, *n* = 5, biological replicates). **u** Apoptosis (annexin V^+^ cells) in EV- or *CTSL* OE-M(IL4) (Student’s *t*-test, ***P < 0.001, *n* = 5, biological replicates). **v** Relative *IL10* mRNA expression in EV- or *CTSL* OE-M(IL4) (Kruskal‒Wallis test, **P < 0.01, *n* = 5, biological replicates). **w** Relative *NRF2* mRNA expression in EV- or *CTSL* OE-M(LPS/IFNγ) and M(IL4) (Kruskal‒Wallis test for M(LPS/IFNγ), Student’s *t*-test for M(IL4), **P < 0.01, *n* = 5, biological replicates). **x** Schematic overview of the proposed mechanism of action for the immunomodulatory effects of NaNO_3_ via the sialin-cathepsin L axis in the liver

To assess *NRF2* expression levels in MASH-affected liver tissue, we examined single-cell sequencing datasets and observed a reduction in *NRF2* mRNA levels within macrophages from individuals with MASH. Furthermore, we found that *SLC17A5*-positive macrophages presented increased mRNA expression levels of the *NRF2*, *ARG1*, and *CD206*^23,36^ (Fig. 8a, b). To validate these results, we cultured human blood macrophages in vitro, after treating with or without NaNO_3_, and induced polarization through LPS/IFN-γ or IL-4. As illustrated in Fig. 8c, d, NaNO_3_ reduced the proportion of CD11C^+^ cells and *INOS* mRNA level in human macrophages stimulated by LPS/IFN-γ. NaNO_3_-induced apoptosis and decreased *TNF* mRNA expression in human macrophages (Fig. 8e). In addition, after NaNO_3_ treatment, the proportion of CD206^+^ cells and *ARG1* and *IL10* mRNA expression were increased, and apoptosis was decreased in IL-4-stimulated human macrophages (Fig. 8f–h). Moreover, NaNO_3_ upregulated *SLC17A5* and *NRF2* expression and downregulated *CTSL* expression in both CD11C^+^ and CD206^+^ human macrophages (Fig. 8i). The efficacy of NaNO_3_ on the cell proportion, apoptosis rate and gene expression was decreased by *SLC17A5* siRNA (Fig. 8j–p, Supplementary Fig. 9a). Furthermore, CTSL OE reversed immunoregulation, with no marked alterations in CD11C^+^ or CD206^+^ macrophages (Fig. 8q–v, Supplementary Fig. 9b). CTSL OE reversed the alteration in *NRF2* mRNA expression (Fig. 8w). These findings show that CTSL is significantly conducive to the functionality in human macrophages. To confirm the effects on human macrophages, as reported in previous research,^37^ we conducted additional experiments in which human macrophage cultures were supplemented with FFA to mimic the localized lipid-rich microenvironment of MASH patients, followed by nitrate administration (Supplementary Fig. 9c). Under FFA conditions, nitrate significantly reduced the proportion of CD11C^+^ macrophages while increasing the level of apoptosis in these cells (Supplementary Fig. 9d, e). Conversely, nitrate increased the proportion of CD206^+^ macrophages and suppressed their apoptosis (Supplementary Fig. 9h, i). The contribution of the CTSL-NRF2 signaling axis to these observed outcomes was confirmed (Supplementary Fig. 9f, g, j, k). These results collectively demonstrated that nitrate retains its immunomodulatory capacity in human macrophages under conditions simulating MASH pathophysiology, even within lipid-overloaded microenvironments.

In summary, we provide convincing evidence that NaNO_3_ regulates immune homeostasis by modulating the inflammatory response of MoMFs via the sialin-CtsL-Nrf2 axis (Fig. 8x).

## Discussion

Our findings indicate that oral NaNO_3_ administration significantly lowers plasma transaminase levels and mitigates severe hepatic inflammation and steatosis in mice challenged with a CDHFD, WD, or MCD dietary regimen, and mitigates WD-induced T2DM-like phenotypes. Our results offer innovative perspectives for the clinical prevention and management of metabolic disorders, encompassing MASLD and T2DM. Mechanistically, NaNO_3_ exerts its protective effects on MASLD and T2DM mainly through rebalancing the numbers and functions of inflammation-promoting and -suppressing MoMFs in the mouse liver, fat, and probably other tissues. In vitro and in vivo treatment with NaNO_3_ downregulated the proportion, survival, and *Tnf*/*TNF* expression of CD11C^+^ MoMFs (pro-inflammatory MoMFs) and increased the relevant parameters of CD206^+^ MoMFs (anti-inflammatory MoMFs). Together, these results demonstrate that NaNO_3_ rebalances MoMFs and maintains immune homeostasis, leading to the amelioration of metabolic disorders.

In our process of treating MASLD with nitrate, we also find that long-term nitrate drinking water can maintain good suppression of the body’s inflammatory response and improve the preventive effects on MASLD. Moreover, through various measurements, we find that long-term nitrate drinking water causes minimal damage to systemic organs, further demonstrating the advantages of nitrate in improving metabolic diseases. Previous reports^38^ have indicated that the therapeutic effect of nitrate on metabolic diseases is limited. However, our further analysis reveal that this limitation is due mainly to the requirement for nitrate treatment of metabolic diseases to be conducted at certain concentrations. We find that 4 mM nitrate in drinking water achieves good therapeutic effects, whereas 2 mM nitrate has limited effects. In contrast, previous reports have used 1 mM nitrate, which does not significantly improve metabolic diseases. Moreover, many metabolic diseases, including T2DM, exhibit sex-specific differences in pathophysiology and treatment response.^39–43^ Consequently, the inclusion of both male and female mice in subsequent research would provide a more complete assessment of NaNO_3_’s potential therapeutic benefits.

While NaNO_3_ reportedly exerts its effects mainly through the nitrate‒nitrite‒NO mechanism, we find that elimination of the main source of NO from oral and enteral symbiotic bacteria using ABX fails to impair the ability of NaNO_3_ to modulate the inflammatory response and survival of macrophages in CDHFD-, WD- and MCD-induced animal models. We also provide evidence that NaNO_3_ directly modulates MoMFs in vitro without impacting NO production. Collectively, our findings shed new light on a novel mechanism by which NaNO_3_ regulates MoMFs.

Further investigation into the mechanism through which nitrate modulates MoMFs revealed that the nitrate transporter sialin is integral to this regulatory process. Knockdown of sialin expression leads to abnormal fat metabolism and impairs the utilization of nitrate, suggesting the important role of sialin in systemic metabolism.^25^ However, our in vitro study did not reveal the protective effect of NaNO_3_ on steatotic hepatocytes, suggesting that NaNO_3_ may not regulate hepatocytes directly through sialin. Our experiments with *Slc17a5* sgRNA two-cell embryo mice demonstrate that NaNO_3_ requires the presence of sialin to exert its regulatory effects on MoMFs to prevent metabolic disorders in murine models. Furthermore, sialin knockdown weakens NaNO_3_ regulation of MoMFs in vitro. Collectively, our findings demonstrate that sialin significantly contributes to the modulation of the immune microenvironment by NaNO_3_.

Sialin may engage in non-canonical functions beyond its well-documented role as an ion transporter, potentially involving intracellular signaling or protein‒protein interactions. Our recent studies demonstrate that sialin, which is traditionally regarded as a plasma membrane transporter, is ubiquitously localized across various organelles, including mitochondria, endosomes, the endoplasmic reticulum, and lysosomes (as shown in *bioRxiv*). The interaction between sialin and Rel, predominantly in the perinuclear region, aligns with the widespread cytosolic localization of sialin and suggests a role in preventing Rel nuclear translocation, potentially modulating transcriptional regulation. These findings expand the functional repertoire of sialin beyond the plasma membrane, with significant implications for intracellular signaling and cellular homeostasis. With respect to the modulation of sialin by nitrate, the nitrate concentration in the sialin-overexpressing group increased but was limited. These findings suggest that nitrate functions primarily as an effector, triggering downstream effects by increasing intracellular sialin levels. Critically, sialin overexpression directly amplifies its function, bypassing upstream regulatory mechanisms to activate downstream signaling cascades in a nitrate-independent manner.

In this study, through gain- and loss-of-function approaches, combining data from transcriptome sequencing analyses, we identify CtsL as a critical element in mediating sialin regulation of the survival and function of MoMFs. CtsL, a multifunctional protease, is critically involved in diverse physiological pathways. Previous investigations have identified a significant association between CtsL activity and macrophage functionality.^44–46^ CtsL OE impairs sialin’s regulatory effects on MoMFs, whereas CtsL downregulation has similar effects on MoMFs as NaNO_3_ treatment does. These findings indicate that CtsL is critically involved in modulating the inflammatory response and polarization of MoMFs under the influence of sialin. Nrf2 plays a vital role in macrophage function. Nrf2 can suppress the macrophage inflammatory response and aid macrophages in developing self-protection against oxidative stress.^47,48^ We also hypothesized that Nrf2 regulates the polarization of macrophages in different directions. Previous studies have extensively emphasized the pivotal role of Nrf2 in regulating different sets of macrophage polarization. Furthermore, we assessed this effect and hypothesized that sialin primarily modulates the polarization of macrophages in diverse directions through Nrf2 regulation.^28,49^ In this study, we find that sialin OE activates the Nrf2 pathway through the sialin-CtsL axis. Knockdown of sialin or CtsL OE inhibits sialin-induced Nrf2 expression in MoMFs. Our results support the notion that sialin regulates MoMF function via the CtsL-Nrf2 pathway.

We acknowledge that this study has several limitations. First, while we clearly demonstrate that oral nitrate attenuates metabolic syndrome-like phenotypes in three well-established metabolic disorder mouse models induced by a CDHFD, MCD, or WD, whether nitrate can display similar effects in humans remains unclear. We hope to conduct a clinical trial to validate whether nitrate can protect against metabolic syndrome in humans in the future. Second, in this study, multiple lines of evidence supporting the notion that nitrate attenuates metabolic disorders by rebalancing CD206^+^/CD11C^+^ polarization and the function of MoMFs are obtained based on our results from mouse studies and in vitro cell experiments. While it is currently challenging for us to obtain human liver samples, it is important to validate our findings in human liver samples from different stages of disease in the future. Third, while our results suggest that sialin regulates MoMFs polarization and function to inhibit MASLD development, because sialin is also expressed in hepatocytes, T cells, and other cell types, we cannot exclude the possibility that sialin regulates polarization and function in the other cell types to contribute at least to its anti-MASLD effects. Establishing a mouse model with sialin-specific knockout in macrophages is crucial for further exploration of how nitrate regulates macrophages and its potential role in preventing metabolic diseases, including MASLD. Finally, in this study, we categorized liver macrophages primarily into bone marrow-derived recruited MoMFs and liver-resident KCs. While this classification simplifies liver macrophage populations, we did not perform detailed subclassification in the MASLD liver. Future studies using techniques such as single-cell sequencing will be needed to more precisely identify the specific macrophage subpopulations affected by nitrate. Moreover, glucagon-like peptide-1 receptor agonists^50^ and other published potential strategies for metabolic dysfunction will be employed as positive controls to elucidate their comparative efficacy against sodium nitrate.

In summary, we provide strong evidence that NaNO_3_ alleviates the homeostasis of MoMFs through the sialin-CtsL-Nrf2 axis to prevent the development and progression of metabolic turbulence. NaNO_3_ could represent a potential therapeutic agent for preventing and managing metabolic disorders, including MASLD and T2DM, in human patients.

## Materials and methods

### Mice

C57BL/6 mice (male, eight-week-old) of matched body weights, as well as CD45.1 congenic mice, were procured from Beijing HFK Bioscience (Beijing, China). Transgenic mice expressing *Slc17a5* sgRNA at the two-cell embryo stage were produced following a previously established method^25^ and maintained within the Laboratory Animal Center at Capital Medical University.

All procedures received approval from both the Animal Care and Use Committee of Capital Medical University and the Ethics Committee of Beijing Friendship Hospital, affiliated with the same institution. Animals were housed under environmentally regulated settings featuring a 12-h alternating light and dark photoperiod.

### Induction of metabolic turbulence

Mice were raised with a normal control diet (NCD), choline-deficient high-fat diet (CDHFD; D05010402, Research Diets, New Brunswick, NJ, USA), methionine/choline-deficient diet (MCD; Cat. 10401, Beijing HFK Bioscience, Beijing, China), or western diet (WD; D12079, Research Diets, New Brunswick, NJ, USA) daily. One week before the diets started, NaNO_3_ was supplemented to the drinking water of the experimental groups, while the control groups received normal drinking water. After 4 weeks (MCD experiment), 16 weeks (CDHFD experiment), or 24 weeks (WD experiment), anesthetized mice were euthanized with subsequent collection of their blood and liver. 4 mM NaNO_3_ supplemented in the drinking water was tested and selected according to a previous study.^15,51^ The food intake, water intake, body weight, and weights of the liver, GAT, and PrAT were recorded.

### Mice treated with broad-spectrum antibiotics (ABX)

To eradicate the microbial community in the mice, another group of C57BL/6 mice was pretreated with ABX, which consisted of vancomycin (Inalco Pharmaceuticals, San Luis Obispo, CA, USA), neomycin (Inalco Pharmaceuticals), ampicillin (Inalco Pharmaceuticals), and metronidazole (Sigma‒Aldrich, St. Louis, MO, USA) added to their drinking water (5 mg of every antibiotic each mouse daily) for 2 weeks. Half of the mice received drinking water with 4 mM NaNO_3_ daily, while the other half had access to normal drinking water. After 1 week, all the mice were fed with MCD daily. After 2 weeks, a single oral ABX gavage was administered to all the mice. Following a subsequent fortnight, mice were euthanized under anesthesia, after which blood and hepatic specimens were obtained.

### Bone marrow-reconstituted mice

CD45.1 congenic C57BL/6 mice (recipient animals) were treated with 900 rad γ-irradiation via the Gammacell® 1000 Elite (Best Theratronics, Springfield, VA, USA). To avoid lethal infections, irradiated mice were given 2 mg/mL neomycin. Donor bone marrow was gained from the tibias and femurs of 8-week-old *Slc17a5* sgRNA two-cell embryo mice or wild-type (WT) mice and resuspended in RPMI 1640 medium (Corning, NY, USA). Bone marrow cells were harvested by flushing via a 22-G needle, followed by filtration via a 40 μm strainer; cells were then resuspended. Each irradiated recipient was transplanted with one injection of 2 × 10^7^ bone marrow cells within one day of irradiation via tail vein injection.

After 4 weeks, half of each recipient group received NaNO_3_ supplementation (4 mM) in their drinking water, while the other half had access to normal drinking water. After bone marrow reconstitution for 5 weeks, all recipient mice were fed an MCD; each group was maintained on NaNO_3_ or the control treatment. After another 4 weeks, following sacrifice, murine blood and hepatic tissue were extracted.

### Plasma, hepatic, and systemic nitrate/nitrite concentrations

An assay kit (KGE 001, R&D Systems, Minneapolis, MN, USA) was utilized. After sacrifice, the plasma (centrifuged at 3500 rpm for 5 min) was moved to another tube at -80 °C. For mouse livers, hearts, muscles, brains, lungs, spleens, kidneys, large intestines, small intestines, PrAT, and GAT, the specific steps were as follows. We first diluted the 10× Reaction Diluent to 1× with ddH_2_O and added a defined volume of 1× Reaction Diluent to the tissue sample. Following complete homogenization, the sample was centrifuged at 12,000 rpm for 10 min. In a 96-well plate, 25 μL NADH, 50 μL standard/sample filtrates, and 25 μL diluted Nitrate Reductase were sequentially supplemented. The mixture was subsequently incubated for a period of 30 min. 50 μL Griess Reagent I and 50 μL Griess Reagent II were then added. Absorbance measurements (OD) were taken at 540 nm after incubating the samples for 10 min, utilizing a reader. A calibration curve was constructed, and corresponding standard concentrations were used to determine nitrate levels in the samples, expressed in μM.

To normalize for tissue mass, supernatant protein levels after centrifugation were detected using a BCA Assay Kit. The diluted standards and samples (20 μL) were loaded into a 96-well plate. A BCA working mixture (Reagent A:B = 50:1) was prepared, and 200 μL was supplemented to each well. Following a 37 °C incubation for 30 min, the OD values were assessed at 562 nm. A standard curve was used to calculate concentrations in mg/mL.

Prior to adding 1× Reaction Diluent, tissue weight was recorded. Nitrate levels were normalized both as μM/(g protein) (nitrate/protein concentration ratio) and μM/(g tissue) (nitrate/tissue weight ratio) to enable cross-comparison across tissue volumes. The experimental results confirmed strong concordance between these two normalization methods.

### Assessment of multiorgan function

The plasma levels of ALT, AST, TC, TG, CK-MB, UA, BUN, CRE, T3, and T4 were analyzed via clinical test kits following the protocol provided by the manufacturer (C009-2, C010-2, A111-1-1, A110-1-1, H197-1-2, C012-2-1, E2020, C011-2-1, H222-1-2, H223-1-2, NJJC Bio, Nanjing, China). Plasma levels of insulin were analyzed via clinical test kits (10-1247-01, Mercodia, Uppsala, Sweden).

### Glucose tolerance test (GTT) and insulin tolerance test (lTT)

To evaluate glucose homeostasis, GTT and ITT were conducted. For the GTT, animals underwent a 16-h fasting period. before receiving glucose at 2 g per kg/body weight. Data were subsequently tracked at multiple time points by collecting blood samples from the tail vein. In the ITT experiment, after a 4-h fasting period, mice on a WD were administered insulin intraperitoneally at a dosage of 1 unit per kg. Subsequent glucose measurements were taken at predetermined time points following insulin injection.

### Measurement of hydroxyproline content

A hydroxyproline assay kit (A030-2-1, NJJC Bio, Nanjing, China) was employed to measure the hydroxyproline levels of the liver samples following the manufacturer’s protocol.

### Monocyte isolation and cultivation from murine bone marrow

Bone marrow was harvested, and red blood cells were then eliminated using a specialized lysis reagent (Cat. 420301, BioLegend, San Diego, CA, USA). To isolate monocytes, cells positive for Gr1 (a granulocyte marker), B220 (a B-cell marker), and Ter119 (an erythroid lineage marker) were depleted through magnetic column-based separation. The purified monocytes were then put in RPMI 1640 with fetal bovine serum (FBS), which provided macrophage colony-stimulating factor (M-CSF) as a conditioning agent. Following a 5-day culture period, the adherent macrophages were transferred to multiwell plates and allowed to settle overnight before stimulation.

In subsequent experiments involving NaNO_3_ exposure, this compound was introduced to the cultured macrophages. After 48 h, the cells were given 100 ng/mL LPS plus 20 ng/mL IFN-γ over a 24-h period to induce an inflammation-promoting phenotype (characterized by CD11C expression). Alternatively, an anti-inflammatory macrophage population (identified by CD206^+^) was generated by 20 ng/mL IL-4.

For studies involving Nrf2 pathway inhibition, ML385 (10 μM; S8790, Selleck, Houston, TX, USA) was administered following NaNO_3_ treatment.

### Isolation of mouse liver immune cells

Following deep anesthesia, a catheter was securely introduced into the portal vein of each mouse and firmly fixed. The inferior vena cava was then incised to permit blood drainage. Hepatic perfusion was initiated using normal saline, which was continued until the liver exhibited a pale appearance. The perfusate was subsequently switched to a pre-warmed enzymatic solution consisting of 1× Hank’s balanced salt solution (HBSS) plus 0.01% collagenase IV (Sigma‒Aldrich, MO, USA), 0.02% bovine serum albumin (BSA; A8806, Sigma‒Aldrich, MO, USA), 0.001% DNase I (Roche, Mannheim, Germany), and 1 mM CaCl_2_. This solution was perfused at 5 mL/min for 15 min while maintained at 37 °C. The organ was then carefully excised and further digested by incubation in fresh enzymatic solution under identical conditions for an additional 15 min to ensure complete tissue dissociation.

Following enzymatic digestion, the liver tissue was gently disaggregated by mechanical means, and the resultant suspension was filtered through a 70-μm nylon filter. Subsequent centrifugation at 50 × g for 5 min separated the mixture into supernatant and sediment fractions. The supernatant underwent additional centrifugation at 500 × g for a duration of 5 min. The resulting cell pellet was then subjected to two washes. Finally, the cells were resuspended in 1× HBSS, carefully layered onto a 30% Percoll gradient (GE Healthcare, Boston, MA, USA), and centrifuged. The pellet obtained after this step consisted primarily of mononuclear cells.

### Isolation and culture of human blood monocytes

Peripheral blood was collected from healthy human donors aged 30–60 years (with no medical history of chronic liver disease, rheumatic immune disease, diabetes mellitus, or malignant tumors) according to the Scripps Research Institute protocol. The donors provided informed consent (CMUSH-IRB-KJ-PT-2024-09), and blood collection was approved by the Institutional Care and Ethics Committee of the Beijing Stomatological Hospital. Human blood monocytes were isolated via the Direct Human Monocyte Isolation Kit (Cat. 19669, STEMCELL Technologies, Cambridge, MA, USA) and cultured in 24-well dishes with RPMI 1640 plus 10% FBS plus 100 ng/mL M-CSF for 7 days for differentiation.

For the experiments with NaNO_3_ treatment, 2 mM NaNO_3_ was used. After 48 h, to induce polarization into pro-inflammatory (CD11C^+^) macrophages, LPS and IFN-γ were supplemented to the macrophage medium for 24 h. Anti-inflammatory (CD206^+^) macrophages were obtained by IL-4 for 24 h.

### Culture of mouse macrophages and normal hepatocyte cells

The RAW264.7 cells were cultured in RPMI 1640 at 37 °C in 5% CO_2_. AML12 cells, a mouse hepatocyte line, were grown in a mixed medium composed of equal parts DMEM and F12-K, supplemented with 1% insulin–transferrin–selenium–ethanolamine (100×; 51500056, Thermo Fisher Scientific, PA, USA), under the same conditions of 37 °C and 5% CO_2_.

### Construction of stable *Slc17a5-*overexpressing macrophages

The overexpression vector system was used to construct a plasmid containing the CDS of *Slc17a5*. *Slc17a5* was integrated into the genome of macrophages (RAW264.7 cells) via transfection, and high-copy number clones with stable expression were obtained by screening with G418 sulfate (Selleck, Houston, TX, USA).

### Western blot analysis

Macrophages were rinsed with PBS. Total protein was gained via RIPA lysis reagent (Solarbio, Beijing, China). Nuclear and cytosolic extracts were isolated by using a commercial kit (P1200-100, Applygen, Beijing, China). Concentrations were quantified with the bicinchoninic acid (BCA) method, following the supplier’s instructions (Yeasen, Shanghai, China). Equivalent quantities of protein (40 μg per lane) were separated electrophoretically on 4–20% SDS‒PAGE gels and subsequently electrotransferred onto PVDF membranes (10600023, GE Healthcare, MA, USA).

The membranes were first incubated with the following primary antibodies: anti-sialin (PA5-30517, Thermo Fisher Scientific; 1:1000), anti-cathepsin L (sc-390367, Santa Cruz Biotechnology; 1:1000), anti-Rel (sc-6955, Santa Cruz Biotechnology; 1:1000), anti-eNOS (32027S, Cell Signaling Technology; 1:500), anti-α-SMA (MA1-06110, Thermo Fisher Scientific; 1:500), anti-H3 (17168-1-AP, ProteinTech; 1:1000), anti-Gapdh (5174T, Cell Signaling Technology; 1:2000), anti-His (12698S, Cell Signaling Technology), and anti-Hsp90 (sc-13119, Santa Cruz Biotechnology; 1:2000). Subsequently, species-matched secondary antibodies (EarthOx, Burlingame, CA, USA; E030120-01 or E030110-01) at a dilution of 1:3000 were used, conjugated to horseradish peroxidase (HRP).

### Real-time quantitative PCR

Total RNA was extracted with an extraction kit (LS1040, Shanghai Promega, China). cDNA was subsequently generated with the PrimeScript RT Reagent Kit (RR037A, Takara Bio, Japan). Real-time PCR was carried out on ABI 7500 (Applied Biosystems, USA). Each amplification reaction, with a total volume of 20 µL (encompassing 10 µL SYBR Green Master Mix, 0.5 µM of both forward and reverse primers, and 1 µL cDNA). The relative fold changes (to *Gapdh* or *GAPDH*) were calculated via the 2^–ΔΔCt^ approach. The sequences of all primers used are provided in Supplementary Table 1.

### CUT&Tag quantitative PCR

The CUT&Tag experiment was conducted using a commercial assay kit (TD903-01, Vazyme, Nanjing, China) in strict accordance with the provided protocol. In brief, MoMFs were harvested and attached to concanavalin A-conjugated magnetic beads. Cells were then treated with a primary antibody targeting Rel (diluted 1:1000; sc-6955, Santa Cruz Biotechnology, USA) or, for control purposes, IgG. Subsequently, the samples were incubated using a corresponding species-specific secondary antibody provided within the kit. Then, the pA-Tn5 transposase complex was introduced to facilitate tagmentation. Genomic DNA was then extracted, amplified, and purified. The final DNA libraries using primers, forward: 5′-CTTAAGACTACCGTGGCGCT-3′, reverse: 5′-TTCCAAGGGCAAGGAAGACC-3′—to identify regions bound by Rel.

### Dual-luciferase reporter assay

The *Ctsl* promoter construct or an empty vector control was introduced into MoMFs together with either Rel mimic or control mimic oligonucleotides, using Lipofectamine 3000 (Invitrogen, CA, USA) as the transfection reagent. Luciferase activity was quantified 48 h following transfection.

### Co-immunoprecipitation (CoIP) and immunoprecipitation-mass spectrometry (IP-MS)

Cells were maintained in 100 mm culture dishes until reaching 95–100% confluency. Following two washes with PBS, cells were harvested by scraping and lysed for 30 min using 1 mL of modified RIPA buffer (Cell Signaling Technology, Danvers, MA, USA), supplemented with inhibitors of proteases and phosphatases (Sigma‒Aldrich, St. Louis, MO, USA). Then it was centrifuged at 10,000 × g for 30 min. To preclear the samples, 50 µL protein G bead was introduced and mixed under rotation overnight. Supernatant was placed into a fresh microcentrifuge tube and combined with 1 µg of an anti-Rel mouse monoclonal antibody (dilution 1:1000; sc-6955X, Santa Cruz Biotechnology, Dallas, TX, USA), followed by rotation overnight. Then, protein G beads were introduced, and the mixture was incubated for another 3–4 h. After three washes with RIPA buffer, the beads were in 95 °C for 5 min in 2× SDS sample buffer. Aliquots of 20 µL (30 µg) were separated by SDS‒PAGE.

Stable His-Slc17a5-expressing Raw264.7 cells were lysed in IP buffer plus 0.3% NP-40 for 40 min. The lysates were incubated with anti-His affinity beads to form an immunoaffinity complex, loaded onto a preequilibrated column. The column was then incubated at 4 °C for 3 h. After binding, the resin was rinsed with IP buffer. Proteins that remained bound were competitively eluted using a medium-concentration His-tag peptide solution. The eluates were collected, denatured in loading buffer, and resolved by NuPAGE SDS‒PAGE (Invitrogen, CA, USA). Proteins were visualized via silver staining (Pierce Silver Staining Kit, Invitrogen, CA, USA) and identified via liquid chromatography‒tandem mass spectrometry.

### Proximity ligation assays (PLA)

Raw264.7 cells were processed following the standard procedure provided with the Duolink® In Situ Detection Reagents Red (DUO92008, Sigma‒Aldrich, MO, USA). Primary antibodies targeting Sialin (diluted at 1:100; PA5-30517, Thermo Fisher Scientific, PA, USA) and CtsL (diluted 1:100; sc-390367, Santa Cruz Biotechnology, Dallas, TX, USA) were incubated with the samples at 4 °C overnight. Subsequently, species-matched PLA probes—specifically Anti-Mouse MINUS and Anti-Rabbit PLUS (Duolink® In Situ PLA® Probe)—were introduced as directed by the manufacturer. Cell nuclei were counterstained using a Duolink® mounting medium containing DAPI. Imaging was carried out with a Nikon confocal microscope system (AX NIS-Elements 5.4, Japan) to acquire fluorescence micrographs.

### Molecular docking

The predicted structural model of proto-oncogene Rel was obtained from the UniProt database (UniProt ID: P15307). Structural models of wild-type sialin and its mutant variant (sialin-AA, with SER17 and GLU14 mutated to alanine) were generated via AlphaFold3.^52^ Rigid-body docking simulations between sialin (wild-type and AA mutant) and Rel were performed via the Z-DOCK server.^53^ Following docking, the predicted complex structures were examined to detect possible binding interfaces and interaction residues. The interface, including hydrogen bonds and contact area, was evaluated via the PDBePISA server (https://www.ebi.ac.uk/pdbe/pisa/). Visualization and distance measurements were conducted via UCSF ChimeraX.^54^

### Histopathological and immunostaining evaluation

Individual murine liver specimens were embedded in paraffin. Then they were rehydrated and stained with H&E or Oil Red O. Each tissue section was evaluated in a blinded manner. H&E-stained sections were semiquantitatively evaluated via the NAS, which is based on semiquantitative scoring of four histological features: steatosis (0–3), lobular inflammation (0–2), hepatocellular ballooning (0–2), and fibrosis (0–4).

Individual adipose tissue specimens obtained from mice were preserved using phosphate-buffered formalin and subsequently embedded in paraffin. Sections of 5 µm thickness were prepared, mounted onto glass slides, and subjected to deparaffinization and rehydration. After staining with H&E, the mean diameter (μm) of adipocytes was evaluated.

For immunohistochemical staining, tissue sections were incubated with an anti-F4/80 primary antibody (diluted at 1:500; GB11027, Servicebio, Wuhan, China) to identify neutrophil populations.

### siRNA and plasmid transfection

Small interfering RNA (siRNA) oligonucleotides targeting *Slc17a5* and *Ctsl*, along with a non-targeting scrambled siRNA control, were designed and synthesized. The specific siRNA sequences employed were: murine *Slc17a5* siRNA (5′-CAAGCCGCTGACTATTTAA-3′), murine *Ctsl* siRNA (5′-GGCTATGAAGGAACAGATT-3′), and human *SLC17A5* siRNA (5′-TCCTGGAGGATATGTTGCCAGCAAA-3′). Transfection of bone marrow-derived monocytes with either gene-specific or control siRNA was performed under Lipofectamine RNAiMAX Transfection (13778150, Thermo Fisher Scientific, PA, USA). Similarly, human peripheral blood monocytes were transfected with *SLC17A5* or scrambled siRNA. At 24 h post-transfection, the cells were given LPS/IFN-γ or IL-4 to promote MoMFs polarization as previously described.

For overexpression studies, plasmid constructs expressing *Ctsl* (pcDNA3.1–3xFLAG-T2A-EGFP) or its corresponding empty vector were procured from GenePharma. An analogous plasmid was used for human *CTSL* overexpression. Monocytes were transiently transfected with the plasmid constructs employing the Lipofectamine 3000 Transfection Kit (L3000015, Thermo Fisher Scientific, PA, USA). Human blood monocytes were similarly transfected with the *CTSL* overexpression plasmid or empty vector. After 24 h, polarized activation of MoMFs was induced through LPS/IFN-γ or IL-4, following the above-mentioned protocol.

### Flow cytometric analysis

The procedures for isolating and analyzing immune cells from both liver and adipose tissues were carried out as reported previously.^55^ Macrophages derived from C57BL/6 mouse bone marrow, RAW264.7 cells, human blood-derived macrophages, and other hepatic immune cell populations were labeled using specific surface or intracellular markers in a staining buffer containing 1.0% BSA. The fluorescently labeled antibodies listed below were acquired from BioLegend, located in San Diego, California, USA: anti-mouse CD3, CD4, CD8, CD11b, CD11C, CD25, CD45.1, CD45.2, CD69, CD206, Ter119, B220, Ly6C, Ly6G, MHC II, NK1.1, TCRβ, TNF-α, Clec4F, and Tim4; as well as anti-human CD14, CD16, CD11C, and CD206. An antibody targeting Sialin (PA5-30517, Thermo Fisher Scientific, PA, USA) was also employed.

Apoptosis was measured by Annexin V-PE detection kit (BD Biosciences, San Jose, CA, USA). Cell viability was determined by staining with 7-aminoactinomycin D (7AAD; BioLegend, San Diego, CA, USA). Transfection efficiency was monitored via fluorescence detection of FAM or GFP signals excited by a 488 nm laser. Lipid droplet accumulation was assessed with Bodipy staining (Invitrogen, CA, USA) following the supplier’s guidelines.

Flow cytometric analysis of all samples was performed using a BD FACS Aria II, and FlowJo (Tree Star, Ashland, OR, USA). The absolute counts of immune cells present in hepatic and adipose tissues were determined based on previously established methods.^56^

### Image flow cytometry

Following PBS washing, cells were resuspended and transferred to a U-bottom plate at a density of 200 µL per well. The mixture was subsequently centrifuged at 300 × g for a duration of 5 min, after which the supernatant was gently aspirated. Cell fixation was performed using 1.5% paraformaldehyde (200 µL per sample) with incubation on ice for 20 min. To enable nuclear staining, samples were permeabilized by slow addition of 70% methanol (200 µL per sample) under gentle agitation, and subsequently kept on ice for 15 min.

An anti-Rel antibody (dilution 1:1000; sc-6955X, Santa Cruz Biotechnology, Dallas, TX, USA) was applied, and samples were incubated overnight. After that, the sections were added with a Hoechst-conjugated secondary antibody (Invitrogen, CA, USA) for 30 min, shielded from light throughout the process. Cellular and subcellular morphological changes, particularly within nuclear and cytoplasmic compartments, were visualized using image flow cytometry (Amnis ImageStream Mk II, Luminex, Austin, TX, USA). Data analysis was conducted using IDEAS software version 6.2.

### Transcriptome sequencing and analysis

Total RNA was extracted, and quality verification was performed on a Bioanalyzer instrument (Agilent, La Jolla, CA, USA). Subsequent library preparation and bioinformatic processing were carried out by the Beijing Genomics Institute (Shenzhen, China). Sequencing was conducted on the BGISEQ-500RS platform provided by the same institute.

### Blinding and control strategies and statistical analysis

Sample acquisition and subsequent data interpretation were carried out by distinct researchers in compliance with a double-blind experimental design. Individual animals or groups were assigned randomly by an independent researcher not involved in behavioral testing or data analysis. Treatment identities were concealed until statistical analysis was complete; personnel performing behavioral tests (rotarod test), tissue processing, or image quantification were unaware of the names of the groups.

All analyses were by GraphPad Prism 9.0 (GraphPad Software, La Jolla, CA, USA). Data are presented as mean ± SEM. Statistical comparisons involving two groups were conducted with Student’s *t*-test for normal distribution data, while the Kruskal–Wallis test was employed for datasets that did not meet normality assumptions. For comparisons among multiple groups, normally distributed data were assessed through one-way ANOVA with subsequent appropriate post hoc analyses, while non-normally distributed data were examined by the Kruskal–Wallis approach. All statistical analyses were conducted with SPSS Statistics 22.0 (IBM, Armonk, NY, USA). For every statistical test performed, a *p*-value of <0.05 was considered significant.

## Supplementary information

SUPPLEMENTAL MATERIAL

Uncropped western blot

## Data Availability

The mRNA sequencing results from this research have been deposited in the Gene Expression Omnibus database at the National Center for Biotechnology (www.ncbi.nlm.nih.gov/geo, GSE204856, GSE248235). All original and uncropped Western blots labeled with molecular weights, sample names, and corresponding figures are available in the Supplementary Materials.
