# Supplementary figures and images for: Sodium nitrate protects against metabolic syndrome by sialin-mediated macrophage rebalance
Source: Signal Transduct Target Ther. 2025 Oct 2;10:323. doi: 10.1038/s41392-025-02418-1 (PMC12488962; doi:10.1038/s41392-025-02418-1)

Uncropped western blots


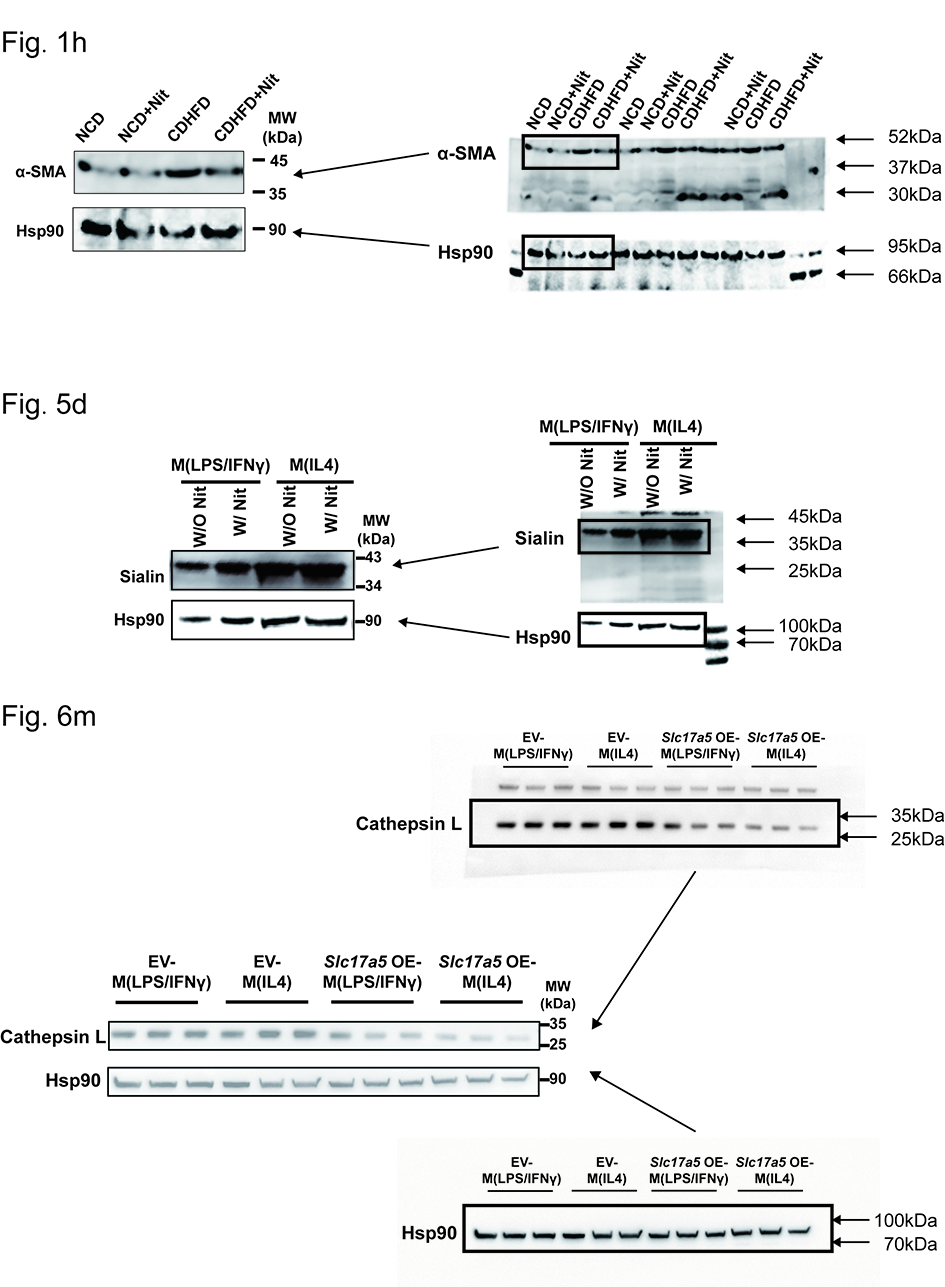


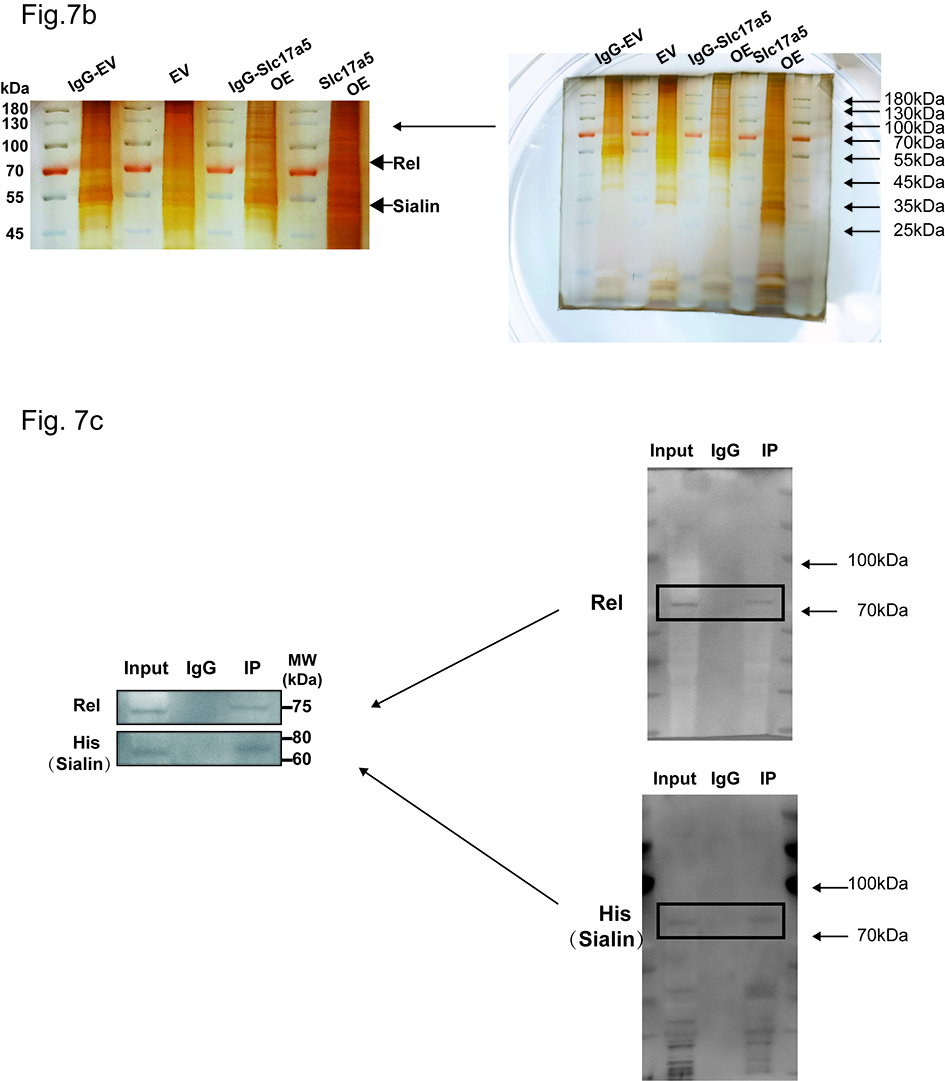


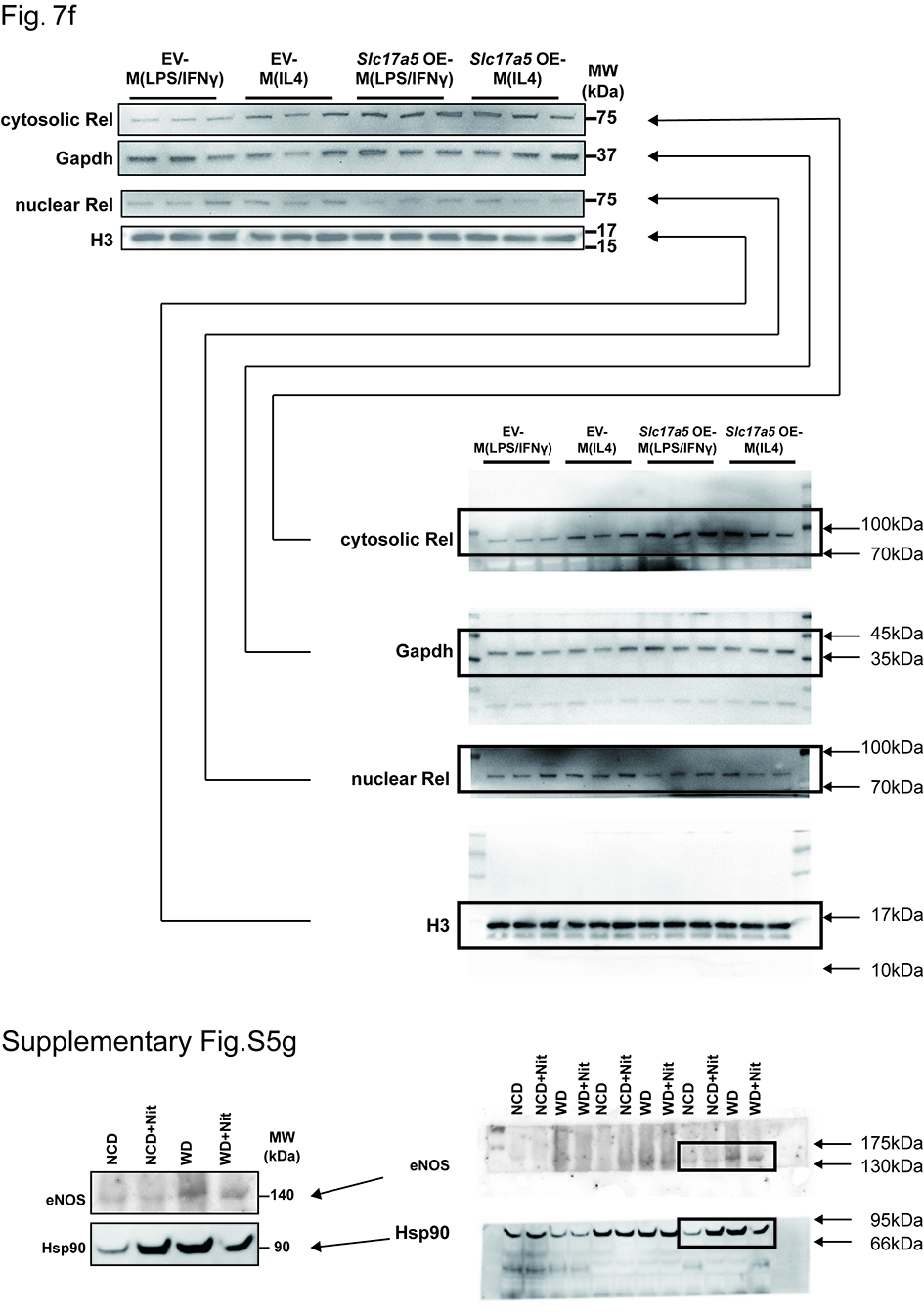


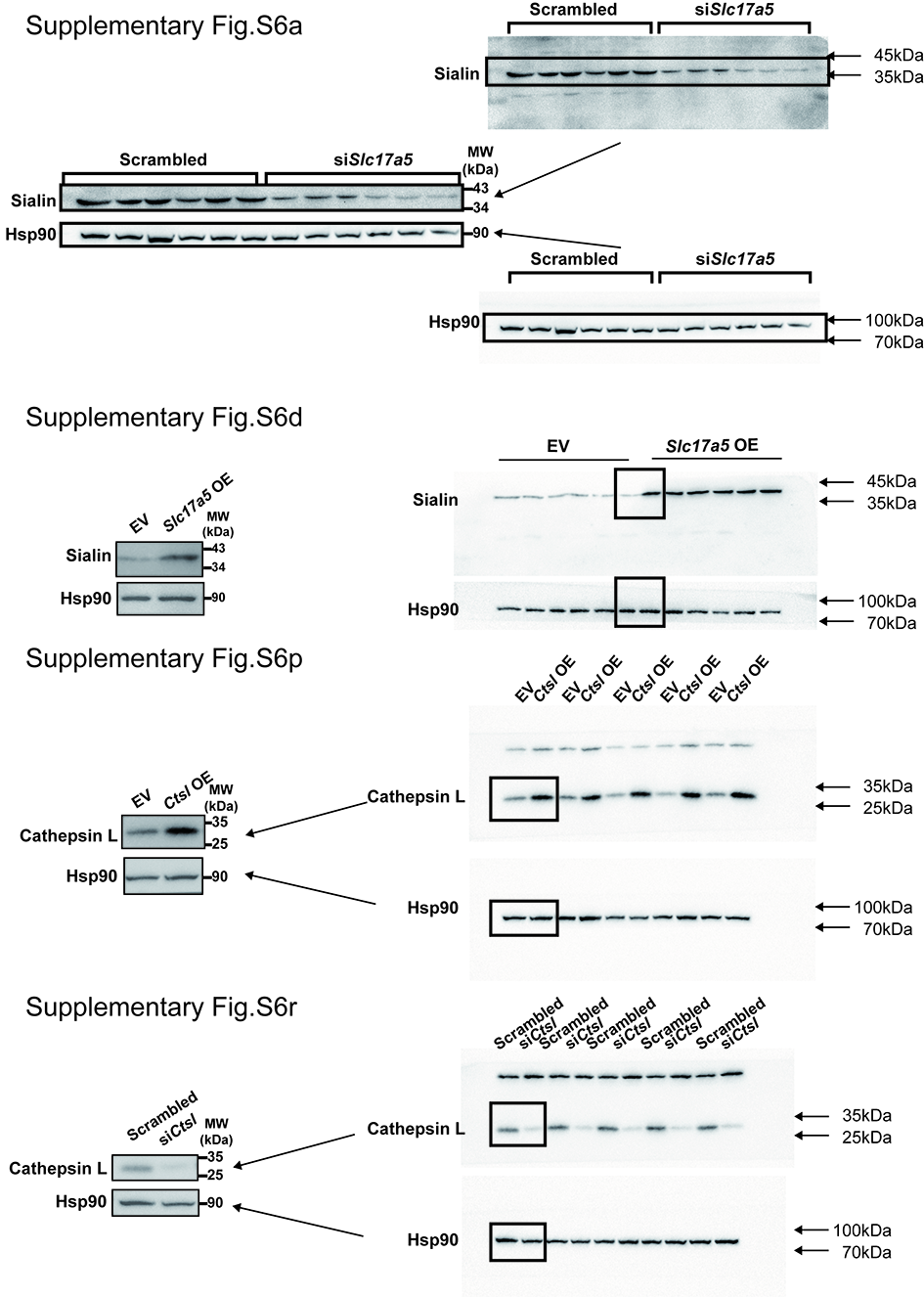


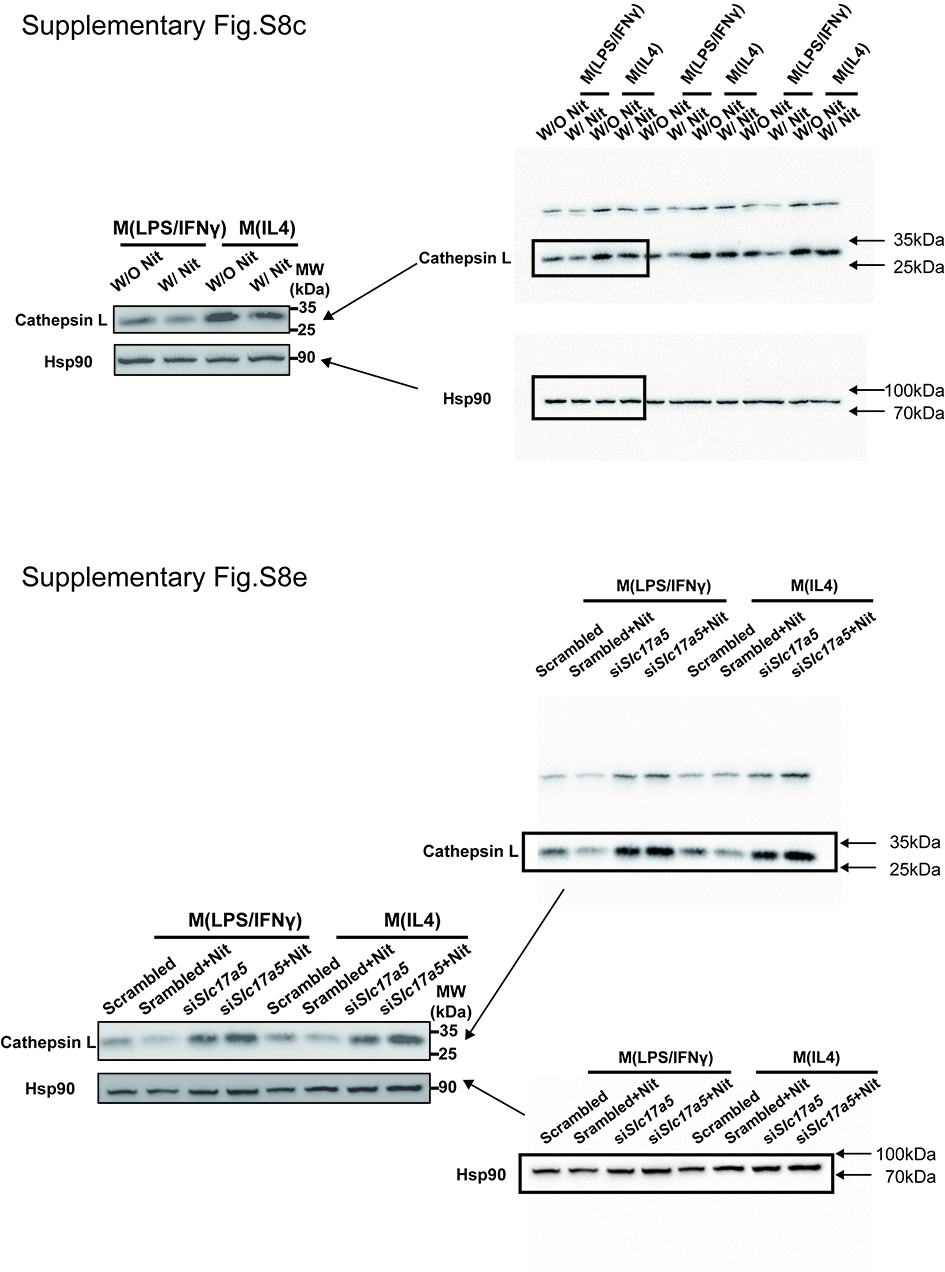

Supplement: Supplementary file 2 — Uncropped western blot [file 41392_2025_2418_MOESM2_ESM.docx]
