# Supplementary material for: Sodium nitrate protects against metabolic syndrome by sialin-mediated macrophage rebalance
Source: Signal Transduct Target Ther. 2025 Oct 2;10:323. doi: 10.1038/s41392-025-02418-1 (PMC12488962; doi:10.1038/s41392-025-02418-1)
Supplement: Supplementary file 1 — SUPPLEMENTAL MATERIAL [file 41392_2025_2418_MOESM1_ESM.docx]

Supplementary Materials for

Sodium nitrate protects against metabolic syndrome by sialin-mediated macrophage rebalance

Shaorong Li, Yaning Wang, Zihan Zhang, Haozhe Xu, Songyue Wu, Hua Jin, Xiaotong Han, Ying Liu, Xin Wen, Yi Wu, Zhongtao Zhang, Lei Hu, Liang Hu, Chunmei Zhang, Jinsong Wang, Renhong Yan, Mo Chen, Guozhi Xiao, Guangyong Sun^*^, Dong Zhang^*^, and Songlin Wang^*^

*Correspondence to: slwang@ccmu.edu.cn, zhangd@ccmu.edu.cn, sungy@ccmu.edu.cn

**This PDF file includes:**

Supplementary Materials and Methods

Supplementary Fig. 1 to 9 (with figure legends below the figures)

Supplementary table 1 (with table legend above the table)

**Other Supplementary Materials for this manuscript include the following:**

Uncropped western blots

Supplementary Materials and Methods

**Rotarod test**

The mice were acclimatized to the testing room for at least 30 minutes prior to the experiment. The rotarod apparatus (Ugo Basile, Italy) was set to accelerate from 4 to 40 rpm over a 300-sec period. Mice were set upon the rotating rod, and the latency to fall (in seconds), latency to latence and speed were recorded.

**Intracell nitrate concentration assay**

Modified liquid chromatography tandem-mass spectrometry (LC‒MS/MS) was used to measure the intracell nitrate concentration in accordance with the following steps.

Cell Preparation: Cells in 100 mm dishes were collected, centrifuged, and washed three times with PBS. The total cell count was determined before the last wash. After centrifugation, the supernatant was removed, and the cells were resuspended in 1 mL of Watsons water. The suspension was centrifuged again, and the cell pellet was collected and stored at -80°C. Preparation for mass spectrometry analysis: the cell pellet was removed from -80°C and subjected to three freeze‒thaw cycles to fully dissolve the target molecules and precipitate proteins. Preparation of sample dilution buffer and standard dilution buffer included the following steps: 10 mL Watsons mineral water + 20 mL LC‒MS grade acetonitrile (A955‒4, Thermo Fisher Scientific, PA, USA) + 200 μL formic acid (F301957‒50 mL, Aladdin, Shanghai, China). The dilution buffer was used to reconstitute the samples and standards. The samples were centrifuged at 12,000 rpm for 15 minutes to remove protein precipitates, and the supernatant was collected. The supernatants of the samples were transferred to vials (51820716, Agilent, CA, USA) for analysis with a 6500 Plus Triple Quadrupole LC‒MS/MS System.

**Intracell total nitrosylation level assay**

The intracell total nitrosylation level (SNO) was analyzed via an enzyme-linked immunosorbent assay (ELISA) kit for mouse S-nitrosothiols (GY-L5190; Shanghai Enzyme-linked Biotechnology, Shanghai, China) according to the manufacturer’s instructions.

**Intracell nitric oxide concentration assay**

The intracell nitric oxide concentrations were tested via a fluorescent probe-based assay (Nitric Oxide Assay Kit with DAF-FM DA (S0020S, Beyotime Biotechnology, Shanghai, China)) according to the manufacturer’s instructions.

**Fluorescent multiplex immunohistochemistry (mIHC), tissue imaging and analysis**

Formalin-fixed, paraffin-embedded tissues were sectioned at 5 μm thickness. The slides were deparaffinized in xylene for 30 minutes and rehydrated in absolute ethyl alcohol for 5 minutes (twice), 95% ethyl alcohol for 5 minutes, and 75% ethyl alcohol for 2 minutes sequentially. The slides were washed with distilled water 3 times. A microwave oven was employed for heat-induced epitope retrieval, and during epitope retrieval, the slides were immersed in boiling EDTA buffer (ZLI-9079, ZSbio, Beijing, China) for 15 min. An antibody mixture/blocking agent from Alpha X Bio was used for blocking. mIHC experiments were performed with an AlphaXPainter X30 (Alpha X Bio, Beijing, China), and the following primary antibodies: anti-CD11C (97585, Cell Signaling Technology, MA, USA), anti-CD206 (ab64693, Abcam, MA, USA), anti-CD11b (ab133357, Abcam, MA, USA), anti-F480 (ab300421, Abcam, MA, USA), anti-CtsL (ab321872, Abcam, MA, USA) and anti-Nrf2 (ER1706-41, Huabio, Hangzhou, China) were used.

All the primary antibodies were incubated for 1 hour at 37°C. The slides were subsequently incubated with Alpha X Ploymer HRP Ms+Rb (Alpha X Bio, Beijing, China) for 10 minutes at 37°C. An Alpha X 7-Color IHC Kit (AXT37100031, Alpha X Bio, Beijing, China) was used for visualization. After each staining cycle, heat-induced epitope retrieval was performed to remove all the antibodies, including primary and secondary antibodies. The slides were counterstained with DAPI for 5 minutes and incubated in Antifade Mounting Medium (I0052; NobleRyder, Beijing, China). ZEISS AXIOSCAN 7 (ZEISS, Oberkochen, Germany) was used to scan the images and HALO software (v3.6; Indica Labs, USA) was employed to analyze the images.

Supplementary Fig.1.

**Supplementary Fig.1 Testing dietary nitrate concentration in CDHFD and MCD diet–fed mice, food and water intake.**

a Schematic overview of experiment. 2 mM NaNO_3_(Nit) or normal water or 4 mM sodium chloride (NaCl, to exclude sodium effect) water was treated 1 week before the modulation, after CDHFD diet was fed for 16 weeks, the mice were sacrificed. The scheme was created with BioRender.
b Plasma ALT levels in CDHFD-fed mice. Data are presented as mean ± SEM (One-way ANOVA with a post hoc test, ***P* < 0.01, *n* = 5, biological replicates for each group).
c Plasma AST levels in CDHFD-fed mice. Data are presented as mean ± SEM (One-way ANOVA with a post hoc test, **P* < 0.05, *n* = 5, biological replicates for each group).
d Plasma nitrate concentration among NCD, NCD+4 mM Nit, CDHFD, CDHFD+ 2 mM Nit and CDHFD+ 4 mM Nit groups. Data are presented as mean ± SEM (One-way ANOVA with a post hoc test, **P* < 0.05, *n* = 5, biological replicates for each group).
e Liver nitrate concentration among NCD, NCD+4 mM Nit, CDHFD, CDHFD+ 2 mM Nit and CDHFD+ 4 mM Nit groups. Data are presented as mean ± SEM (One-way ANOVA with a post hoc test, ***P* < 0.01, *n* = 5, biological replicates for each group).

**f-h** Representative H&E-stained liver sections plus graph showing NAS. And representative Oil Red O–stained liver sections plus graph showing semiquantitative analysis of lipid accumulation score in CDHFD-fed mice, Scale bars: 100 μm. Data are presented as mean ± SEM (Kruskal–Wallis test for NAS*,* One-way ANOVA with a post hoc test for lipid accumulation score, ***P* < 0.01, ****P* < 0.001, *n* = 5, biological replicates for each group).

i Schematic overview of experiment. 2 mM nitrate or normal water or 4 mM sodium chloride (NaCl, to exclude sodium effect) water was treated 1 week before the modulation, after MCD diet was fed for 4 weeks, the mice were sacrificed. The scheme was created with BioRender.
j Plasma ALT levels in MCD-fed mice. Data are presented as mean ± SEM (Kruskal–Wallis test, ****P* < 0.001, *n* = 5, biological replicates for each group).
k Plasma AST levels in MCD-fed mice. Data are presented as mean ± SEM (Kruskal–Wallis test, NS no significance, *n* = 5, biological replicates for each group).
l Plasma nitrate concentration among NCD, NCD+4 mM Nit, MCD, MCD+ 2 mM Nit and MCD+ 4 mM Nit groups. Data are presented as mean ± SEM (One-way ANOVA with a post hoc test, ****P* < 0.001, *n* = 5, biological replicates for each group).
m Liver nitrate concentration among NCD, NCD+4 mM Nit, MCD, MCD+ 2 mM Nit and MCD+ 4 mM Nit groups. Data are presented as mean ± SEM (One-way ANOVA with a post hoc test, ****P* < 0.001, *n* = 5, biological replicates for each group).

**n-p** Representative H&E-stained liver sections plus graph showing NAS and representative Oil Red O–stained liver sections plus graph showing semiquantitative analysis of lipid accumulation score in MCD-fed mice, Scale bars: 100 μm. Data are presented as mean ± SEM (Kruskal–Wallis test, NS no significance, *n* = 5, biological replicates for each group).

Supplementary Fig.2.

**Supplementary Fig.2 Effects of NaNO_3_ treatment in CDHFD-fed and MCD-fed mice.**

**a,b** C57BL/6 mice received drinking water with 4 mM NaNO_3_ (Nit) or normal drinking water. After 1 week, half of each group were fed choline-deficient, high-fat diet (CDHFD) and the other half normal control diet (NCD) for 16 weeks, after which all mice were sacrificed and plasma and livers were collected.

a The general water intake of CDHFD-diet prevented groups. Data are presented as mean ± SEM (Kruskal–Wallis test, *n* = 5, biological replicates for each group).
b The general food intake of CDHFD-diet prevented groups. Data are presented as mean ± SEM (Kruskal–Wallis test, *n* = 5, biological replicates for each group).

**c-n** Another group of C57BL/6 mice received drinking water with 4 mM NaNO_3_(Nit) or normal drinking water. After 1 week, half of each group were fed methionine/choline-deficient diet (MCD) and the other half NCD for 4 weeks, after which all mice were sacrificed and plasma and livers were collected.

c Schematic overview of experiment in NCD- and MCD-fed mice. The scheme was created with BioRender.
d The general water intake of NCD- and MCD-fed mice. Data are presented as mean ± SEM (One-way ANOVA with a post hoc test, *n* = 5, biological replicates for each group).
e Nitrate concentrations in plasma of NCD- and MCD-fed mice. Data are presented as mean ± SEM (Kruskal–Wallis test, **P* < 0.05, ***P* < 0.01, *n* = 5, biological replicates for each group).
f Nitrate concentrations in liver of NCD- and MCD-fed mice. Data are presented as mean ± SEM (One-way ANOVA with a post hoc test, ***P* < 0.01, ****P* < 0.001, *n* = 5, biological replicates for each group).
g Plasma ALT levels in NCD- and MCD-fed mice. Data are presented as mean ± SEM (One-way ANOVA with a post hoc test, ****P* < 0.001, *n* = 5, biological replicates for each group).
h Plasma AST levels in NCD- and MCD-fed mice. Data are presented as mean ± SEM (Kruskal–Wallis test, ***P* < 0.01, *n* = 5, biological replicates for each group).

**i-k** NAS and semiquantitative score of Oil Red O staining. Representative H&E-stained liver sections and representative Oil Red O–stained mouse liver sections. Scale bars: 100 μm. Data are presented as mean ± SEM (One-way ANOVA with a post hoc test, ****P* < 0.001, *n* = 5, biological replicates for each group).

l Relative mRNA expression of fibrosis-related genes *Col1a1*, *Col1a3*, and *Acta2* mRNA in mouse livers in NCD- and MCD-fed mice. Data are presented as mean ± SEM (Kruskal–Wallis test for *Col1a3* and *Acta2,* One-way ANOVA with a post hoc test for *Col1a1*, **P* < 0.05, ***P* < 0.01, *n* = 5, biological replicates for each group).
m Relative mRNA expression of pro-inflammatory and anti-inflammatory cytokine factors in mouse livers in NCD- and MCD-fed mice. Data are presented as mean ± SEM (Kruskal–Wallis test for *Tgfb1, Ifng* and *Il13,* One-way ANOVA with a post hoc test for others, ***P* < 0.01, ****P* < 0.001, *n* = 5, biological replicates for each group).
n Hydroxyproline content in mouse livers in NCD- and MCD-fed mice. Data are presented as mean ± SEM (One-way ANOVA with a post hoc test, **P* < 0.05, ***P* < 0.01, *n* = 5, biological replicates for each group).

Supplementary Fig.3.

**Supplementary Fig.3 Effects of NaNO_3_ treatment in prolonged WD feeding mice.**

**a-p** Two groups of C57BL/6 mice were fed western diet (WD) and the other groups normal control diet (NCD) for 14 months. One week before that, the mice received drinking water with 4 mM NaNO_3_ (Nit) or normal drinking water and remained for 14 months. After which all mice were sacrificed and plasma and livers were collected.

**a** Glucose tolerance test and relative area under the curve value (AUC) in prolonged NCD- and WD-fed mice. Data are presented as mean ± SEM (Kruskal–Wallis test, **P* < 0.05, *n* = 3, biological replicates for each group).

**b** Insulin tolerance test and relative area under the curve value (AUC) in prolonged NCD- and WD-fed mice. Data are presented as mean ± SEM (Student’s *t* test, **P* < 0.05, *n* = 3, biological replicates for each group).

c The body weight, weight of liver and weight of gonadal adipose tissue (GAT) after the experiment in prolonged NCD- and WD-fed mice. Data are presented as mean ± SEM (One-way ANOVA with a post hoc test, **P* < 0.05, ***P* < 0.01, ****P* < 0.001, *n* = 5, biological replicates for each group).
d The level of insulin in prolonged NCD- and WD-fed mice. Data are presented as mean ± SEM (One-way ANOVA with a post hoc test, **P* < 0.05, ***P* < 0.01, *n* = 5, biological replicates for each group).
e Plasma alanine aminotransferase (ALT) level in prolonged NCD- and WD-fed mice. Data are presented as mean ± SEM (One-way ANOVA with a post hoc test, ****P* < 0.001, *n* = 5, biological replicates for each group).
f Plasma aspartate aminotransferase (AST) level in prolonged NCD- and WD-fed mice. Data are presented as mean ± SEM (One-way ANOVA with a post hoc test, ***P* < 0.01, ****P* < 0.001, *n* = 5, biological replicates for each group).
g The level of plasma total cholesterol (TG) in prolonged NCD- and WD-fed mice. Data are presented as mean ± SEM (One-way ANOVA with a post hoc test, ****P* < 0.001, *n* = 5, biological replicates for each group).
h The level of plasma triglycerides (TC) in prolonged NCD- and WD-fed mice. Data are presented as mean ± SEM (Kruskal–Wallis test, ***P* < 0.01, *n* = 5, biological replicates for each group).
i The level of plasma creatine kinase isoenzymes (CK-MB) in prolonged NCD- and WD-fed mice. Data are presented as mean ± SEM (Kruskal–Wallis test, ***P* < 0.01, *n* = 5, biological replicates for each group).
j The level of plasma uric acid (UA) in prolonged NCD- and WD-fed mice. Data are presented as mean ± SEM (One-way ANOVA with a post hoc test, *n* = 5, biological replicates for each group).
k The level of plasma blood urea nitrogen (BUN) in prolonged NCD- and WD-fed mice. Data are presented as mean ± SEM (One-way ANOVA with a post hoc test, *n* = 5, biological replicates for each group).
l The level of plasma creatinine (CRE) in prolonged NCD- and WD-fed mice. Data are presented as mean ± SEM (One-way ANOVA with a post hoc test, *n* = 5, biological replicates for each group).
m The level of plasma triiodothyronine (T3) and thyroxine (T4) in prolonged NCD- and WD-fed mice. Data are presented as mean ± SEM (Kruskal–Wallis test for T3, One-way ANOVA with a post hoc test for T4, *n* = 5, biological replicates for each group).
n Latency to fall of prolonged NCD- and WD-fed mice. Data are presented as mean ± SEM (One-way ANOVA with a post hoc test, *n* = 5, biological replicates for each group).
o Latence of prolonged NCD- and WD-fed mice. Data are presented as mean ± SEM (One-way ANOVA with a post hoc test, *n* = 5, biological replicates for each group).
p Speed of prolonged NCD- and WD-fed mice. Data are presented as mean ± SEM (Kruskal–Wallis test, *n* = 5, biological replicates for each group).
q Nitrate concentrations in heart of prolonged NCD- and WD-fed mice. Data are presented as mean ± SEM (Kruskal–Wallis test, **P* < 0.05, ***P* < 0.01, *n* = 5, biological replicates for each group).
r Nitrate concentrations in muscle of prolonged NCD- and WD-fed mice. Data are presented as mean ± SEM (Kruskal–Wallis test, **P* < 0.05, *n* = 5, biological replicates for each group).
s Nitrate concentrations in brain of prolonged NCD- and WD-fed mice. Data are presented as mean ± SEM (Kruskal–Wallis test, *n* = 5, biological replicates for each group).
t Nitrate concentrations in lung of prolonged NCD- and WD-fed mice. Data are presented as mean ± SEM (Kruskal–Wallis test, ***P* < 0.01, *n* = 5, biological replicates for each group).
u Nitrate concentrations in spleen of prolonged NCD- and WD-fed mice. Data are presented as mean ± SEM (Kruskal–Wallis test, ***P* < 0.01, *n* = 5, biological replicates for each group).
v Nitrate concentrations in kidney of prolonged NCD- and WD-fed mice. Data are presented as mean ± SEM (One-way ANOVA with a post hoc test, **P* < 0.05, ***P* < 0.01, *n* = 5, biological replicates for each group).
w Nitrate concentrations in large intestine of prolonged NCD- and WD-fed mice. Data are presented as mean ± SEM (One-way ANOVA with a post hoc test, **P* < 0.05, *n* = 5, biological replicates for each group).
x Nitrate concentrations in small intestine of prolonged NCD- and WD-fed mice. Data are presented as mean ± SEM (One-way ANOVA with a post hoc test, **P* < 0.05, *n* = 5, biological replicates for each group).
y Nitrate concentrations in perirenal adipose tissue (PrAT) of prolonged NCD- and WD-fed mice. Data are presented as mean ± SEM (Kruskal–Wallis test, **P* < 0.05, *n* = 5, biological replicates for each group).
z Nitrate concentrations in gonadal adipose tissue (GAT) of prolonged NCD- and WD-fed mice. Data are presented as mean ± SEM (Kruskal–Wallis test, **P* < 0.05, *n* = 5, biological replicates for each group).

Supplementary Fig.4.

**Supplementary Fig.4** **Representative flow cytometric images of gating strategy used for flow cytometric analysis and immunoregulation in MCD&CDHFD&&WD-fed mice of NaNO_3._**

a Typical flow cytometry gating strategy used to distinguish hepatic lymphocytes.
b Typical flow cytometry gating strategy used to distinguish hepatic macrophages and Kupffer cells.
c Representative flow cytometric plots of the proportion of resident Kupffer cells (res KCs, Tim4^+^Clec4F^+^) in liver of each group of NCD- or WD-fed mice. Data are presented as mean ± SEM (One-way ANOVA with a post hoc test, *n* = 5, biological replicates for each group).

**d-f** Fluorescent multiplex immunohistochemistry images on liver tissue of CD11C^+^ and CD206^+^ macrophages and semi quantitative analysis. Data are presented as mean ± SEM (Kruskal–Wallis test for CD11C^+^*,* One-way ANOVA with a post hoc test for CD206^+^, **P* < 0.05, ***P* < 0.01, *n* = 3, biological replicates for each group).

g Representative flow cytometric plots of the proportion of Kupffer cells (KCs), natural killer cells (NKs), NK T cells (NKTs) in liver of each group of normal control diet (NCD)- or methionine/choline-deficient diet (MCD)-fed mice. Data are presented as mean ± SEM (Kruskal–Wallis test for NKTs*,* One-way ANOVA with a post hoc test for KCs, NKs **P* < 0.05, *n* = 5, biological replicates for each group).
h Absolute cell numbers of Kupffer cells (KCs), natural killer cells (NKs), NK T cells (NKTs), in liver of normal control diet (NCD)- and methionine/choline-deficient diet (MCD)-fed mice. Data are presented as mean ± SEM (One-way ANOVA with a post hoc test, **P* < 0.05, *n* = 5, biological replicates for each group).
i Representative flow cytometry plots of the proportion of CD11C^+^ MoMFs and CD206^+^ MoMFs among liver in each group of normal control diet (NCD)- or methionine/choline-deficient diet (MCD)-fed mice.
j Representative flow cytometric plots of proportion of TNF-α positive CD11C^+^ MoMFs in mouse livers of normal control diet (NCD)- or methionine/choline-deficient diet (MCD)-fed mice.

Supplementary Fig.5.

**Supplementary Fig.5 The chosen of NaNO_3_ concentration and analysis of eNOS, intracellular nitrate, total nitrosylation and nitric oxide.**

a Bone marrow–derived monocytes were harvested from C57BL/6 mice, treated with 0, 50 μM, 100 μM, 200 μM, 500 μM, 1 mM, 2 mM, 4 mM and 6 mM NaNO_3_ for 48 hours, and then induced *in vitro* to differentiate into MoMFs, polarized into LPS/IFN-γ–stimulated MoMFs (M(LPS/IFNγ)). The scheme was created with BioRender.
b Proportion of CD11C^+^ cells in M(LPS/IFNγ). Data are presented as mean ± SEM (One-way ANOVA with a post hoc test, **P* < 0.05, ***P* < 0.01, ****P* < 0.001, *n* = 5, biological replicates for each group).
c Apoptosis (Annexin V^+^ cells) in M(LPS/IFNγ). Data are presented as mean ± SEM (One-way ANOVA with a post hoc test, **P* < 0.05, ***P* < 0.01, ****P* < 0.001, *n* = 5, biological replicates for each group).
d Bone marrow–derived monocytes were harvested from C57BL/6 mice, treated with 0, 50 μM, 100 μM, 200 μM, 500 μM, 1 mM, 2 mM, 4 mM and 6 mM NaNO_3_ for 48 hours, and then induced to differentiate into MoMFs, polarized into IL-4–stimulated MoMFs (M(IL4)) *in vitro*. The scheme was created with BioRender.
e Proportion of CD206^+^ cells in M(IL4). Data are presented as mean ± SEM (One-way ANOVA with a post hoc test, **P* < 0.05, ***P* < 0.01, ****P* < 0.001, *n* = 5, biological replicates for each group).
f Apoptosis (Annexin V^+^ cells) in M(IL4). Data are presented as mean ± SEM (One-way ANOVA with a post hoc test, **P* < 0.05, ***P* < 0.01, *n* = 5, biological replicates for each group).

**g,h** Relative expression of eNOS protein (normalized for Hsp90 expression). Data are presented as mean ± SEM (Kruskal–Wallis test, *n* = 5, biological replicates for each group).

i Intracell concentration of nitrate. Data are presented as mean ± SEM (Student’s *t* test, **P* < 0.05, *n* = 4 or 5, biological replicates for each group).
j Intracell concentration of total nitrosylation (SNO). Data are presented as mean ± SEM (Student’s *t* test, *n* = 12, biological replicates for each group).
k Intracell concentration of nitric oxide (NO). Data are presented as mean ± SEM (Student’s *t* test, *n* = 8, biological replicates for each group).

Supplementary Fig.6.

**Supplementary Fig.6 Supplementary of modulation by sialin, CtsL and Nrf2 and transcriptome analysis of *Slc17a5* overexpression vs empty vector.**

**a-c** Relative sialin protein expression, *Slc17a5* mRNA expression and mean fluorescence intensity (MFI, transfection efficiency) of bone marrow–derived monocytes, which were harvested from C57BL/6 mice were transfected with *Slc17a5* or scrambled siRNA (control) *in vitro.* Data are presented as mean ± SEM (Kruskal–Wallis test for mRNA, Student’s *t* test for protein and MFI, ***P* < 0.01, ****P* < 0.001, *n* = 6, biological replicates for each group).

**d-f** Macrophages cells were transfected with *Slc17a5* overexpression plasmid (OE) or empty vector (EV) *in vitro*.

d Relative sialin protein expression (normalized for Hsp90 expression) after *Slc17a5* overexpression. Data are presented as mean ± SEM (Student’s *t* test, ****P* < 0.001, *n* = 5, biological replicates for each group).
e Microcopy of transfected GFP cells after *Slc17a5* overexpression and EV (with GFP) transfected.
f Mean fluorescence intensity (MFI, transfection efficiency) after *Slc17a5* overexpression and EV (no GFP) transfected. Data are presented as mean ± SEM ((Student’s *t* test, ****P* < 0.001, *n* = 6, biological replicates for each group).
g Intracell concentration of nitrate. Data are presented as mean ± SEM (One-way ANOVA with a post hoc test, *n* = 4 or 5, biological replicates for each group).
h Intracell concentration of total nitrosylation (SNO). Data are presented as mean ± SEM (Kruskal–Wallis test, *n* = 12, biological replicates for each group).
i Intracell concentration of nitric oxide (NO). Data are presented as mean ± SEM (One-way ANOVA with a post hoc test, *n* = 8, biological replicates for each group).
j mRNA alteration in overexpression sialin of macrophages (*Slc17a5* OE) or empty vector (EV) stimulated with LPS/IFN-γ identified by RNA sequencing.

**k,l** Analysis strategy. We screened differentially expressed genes in CD11C^+^ or CD206^+^ bone marrow monocyte–derived macrophages transfected with *Slc17a5* vector or empty vector (q <0.05). There 8438 differentially expressed genes in bone marrow CD11C^+^ group and 6554 in CD206^+^ group. Considering the regulation of sialin on both CD11C^+^ and CD206^+^ of NaNO_3_, we chose to analyze the subset of co-occurring 4799 genes (shown in Venn diagram).

m Relative *Fas* mRNA expression in M(LPS/IFNγ) and M(IL4) with *Slc17a5* OE or EV. Data are presented as mean ± SEM (Kruskal–Wallis test, *n* = 5, biological replicates for each group).
n Relative *Trp53* mRNA expression in M(LPS/IFNγ) and M(IL4) with *Slc17a5* OE or EV. Data are presented as mean ± SEM (Kruskal–Wallis test, *n* = 5, biological replicates for each group).
o Relative *Pik3cd* mRNA expression in M(LPS/IFNγ) and M(IL4) with *Slc17a5* OE or EV. Data are presented as mean ± SEM (Kruskal–Wallis test, **P* < 0.05, *n* = 5, biological replicates for each group).
p Relative cathepsin L protein expression of bone marrow–derived monocytes, which were harvested from C57BL/6 mice were transfected with *Ctsl* overexpression plasmid (OE) or empty vector (EV) *in vitro*. Data are presented as mean ± SEM (Student’s *t* test, ****P* < 0.001, *n* = 5, biological replicates for each group).
q Mean fluorescence intensity (MFI, transfection efficiency) after *Ctsl* overexpression and EV (no GFP) transfected. Data are presented as mean ± SEM (Student’s *t* test, ****P* < 0.001, *n* = 6, biological replicates for each group).

**r-x** Bone marrow–derived monocytes were harvested from C57BL/6 mice transfected with *Ctsl* siRNA or scrambled siRNA (control) *in vitro*, and then induced to differentiate into MoMFs, polarized into LPS/IFN-γ–stimulated MoMFs (M(LPS/IFNγ)) or IL-4–stimulated MoMFs (M(IL4)).

r Relative cathepsin L protein expression of bone marrow–derived monocytes, which were harvested from C57BL/6 mice, were transfected with *Ctsl* or scrambled siRNA (control) *in vitro*. Data are presented as mean ± SEM (Student’s *t* test, ****P* < 0.001, *n* = 5, biological replicates for each group).
s Mean fluorescence intensity (MFI, transfection efficiency) after *Ctsl* (with FAM) or scrambled siRNA transfected. Data are presented as mean ± SEM (Kruskal–Wallis test, ****P* < 0.001, *n* = 6, biological replicates for each group).
t Proportion of CD11C^+^ cells after *Ctsl* or scrambled siRNA transfected. Data are presented as mean ± SEM (Student’s *t* test, ****P* < 0.001, *n* = 5, biological replicates for each group).
u Relative *Inos* mRNA expression after *Ctsl* or scrambled siRNA transfected. Data are presented as mean ± SEM (Student’s *t* test, ***P* < 0.01, *n* = 5, biological replicates for each group).
v Proportion of CD206^+^ cells after *Ctsl* or scrambled siRNA transfected. Data are presented as mean ± SEM (Student’s *t* test, **P* < 0.05, *n* = 5, biological replicates for each group).
w Relative *Arg1* mRNA expression after *Ctsl* or scrambled siRNA transfected. Data are presented as mean ± SEM (Kruskal–Wallis test*,* ***P* < 0.01, *n* = 5, biological replicates for each group).
x Relative *Nrf2* mRNA expression after *Ctsl* or scrambled siRNA transfected. Data are presented as mean ± SEM (Student’s *t* test, ***P* < 0.01, *n* = 5, biological replicates for each group).

Supplementary Fig.7.

**Supplementary Fig.7 Transcriptome analysis of genes playing key role in sialin’s regulation of MoMFs inflammatory response and survival.**

**a,b** Analysis strategy. We screened differentially expressed genes in CD11C^+^ bone marrow monocyte–derived macrophages (MoMFs) with or without 2 mM NaNO_3_ (q <0.05) and after *Slc17a5* knockdown. 812 differentially expressed genes in CD11C^+^ bone marrow monocyte–derived macrophages (MoMFs) group and 1409 in CD206^+^ bone marrow monocyte–derived macrophages (MoMFs) were detected. Considering the regulation of NaNO_3_ on both CD11C^+^ and CD206^+^ bone marrow monocyte–derived macrophages (MoMFs), we chose to analyze the subset of co-occurring 242 genes (shown in Venn diagram).

**c,d** Genes identified with GO and KEGG pathway analyses showed high enrichment of cell activation, cell death, immune response, inflammatory response, and apoptosis.

Supplementary Fig.8.

**Supplementary Fig.8 NaNO_3_ downregulates CtsL expression and activates Nrf2 signaling in MoMFs.**

a Analysis strategy. 242 genes were enriched, and those related to apoptosis, immune response and lymphocyte activation were selected based on the closeness of association with the function of NaNO_3_ revealed in this study and found *Ctsl* was located at intersection of these 3 areas*.*

**b,c,** Bone marrow–derived monocytes were harvested from C57BL/6 mice, treated with or without 2 mM NaNO_3_, and then induced *in vitro* to differentiate into MoMFs, polarized into LPS/IFN-γ–stimulated MoMFs (M(LPS/IFNγ)) or IL-4–stimulated MoMFs (M(IL4)).

b Relative *Ctsl* mRNA expression in M(LPS/IFNγ) and M(IL4) with or without nitrate. Data are presented as mean ± SEM (Kruskal–Wallis test, **P* < 0.05, ***P* < 0.01, *n* = 5, biological replicates for each group).
c Relative expression of cathepsin L protein (normalized for Hsp90 expression) in M(LPS/IFNγ) and M(IL4) with or without nitrate. Data are presented as mean ± SEM (One-way ANOVA with a post hoc test, ***P* < 0.01, ****P* < 0.001, *n* = 5, biological replicates for each group).

**d,e** Bone marrow–derived monocytes were harvested from C57BL/6 mice, treated with or without 2 mM NaNO_3_, and then transfected with *Slc17a5* or scrambled siRNA (control) *in vitro*, induced to differentiate into monocyte-derived macrophages (MoMFs), polarized into LPS/IFN-γ–stimulated MoMFs (M(LPS/IFNγ)) or IL-4–stimulated MoMFs (M(IL4)).

d Relative *Ctsl* mRNA expression after transfected with *Slc17a5* or scrambled siRNA. Data are presented as mean ± SEM (One-way ANOVA with a post hoc test, **P* < 0.05, *n* = 5, biological replicates for each group).
e Relative expression of cathepsin L protein (normalized for Hsp90 expression) after transfected with *Slc17a5* or scrambled siRNA. Data are presented as mean ± SEM (One-way ANOVA with a post hoc test, ***P* < 0.01, ****P* < 0.001, *n* = 5, biological replicates for each group).

**f,g** Bone marrow–derived monocytes were harvested from C57BL/6 mice, treated with or without 2 mM NaNO_3_, and then transfected with *Ctsl* overexpression plasmid (OE) or empty vector (EV) *in vitro*, induced to differentiate into MoMFs, polarized into LPS/IFN-γ–stimulated MoMFs (M(LPS/IFNγ)) or IL-4–stimulated MoMFs (M(IL4)).

f Proportion of CD11C^+^ cells after transfected with *Ctsl* OE or EV. Data are presented as mean ± SEM (Student’s *t* test, ****P* < 0.001, *n* = 5, biological replicates for each group).
g Relative *Inos* mRNA expression after transfected with *Ctsl* OE or EV. Data are presented as mean ± SEM (Student’s *t* test, ****P* < 0.001, *n* = 5, biological replicates for each group).
h Proportion of CD206^+^ cells after transfected with *Ctsl* OE or EV. Data are presented as mean ± SEM (Student’s *t* test, ***P* < 0.01, *n* = 5, biological replicates for each group).
i Relative *Arg1* mRNA expression after transfected with *Ctsl* OE or EV. Data are presented as mean ± SEM (Kruskal–Wallis test, ***P* < 0.01, *n* = 5, biological replicates for each group).
j Relative *Nrf2* mRNA expression after transfected with *Ctsl* OE or EV. Data are presented as mean ± SEM (One-way ANOVA with a post hoc test, **P* < 0.05, ***P* < 0.01, *n* = 5, biological replicates for each group).

**k,l** Bone marrow–derived monocytes were harvested from C57BL/6 mice, treated with or without 2 mM NaNO_3_ (Nit), and then treated with or without Nrf2 inhibitor (ML385) *in vitro*, induced to differentiate into monocyte-derived macrophages (MoMFs), polarized into LPS/IFN-γ–stimulated MoMFs (M(LPS/IFNγ)) or IL-4–stimulated MoMFs (M(IL4)).

k Proportion of CD11C^+^ cells after treated with or without Nrf2 inhibitor (ML385). Data are presented as mean ± SEM (One-way ANOVA with a post hoc test, **P* < 0.05, *n* = 5, biological replicates for each group).
l Proportion of CD206^+^ cells after treated with or without Nrf2 inhibitor (ML385). Data are presented as mean ± SEM (One-way ANOVA with a post hoc test, **P* < 0.05, *n* = 5, biological replicates for each group).
m Bone marrow–derived monocytes treated with 2 mM NaNO_3_, and then transfected with *Slc17a5* siRNA or scrambled siRNA *in vitro*, induced to differentiate into MoMFs, polarized into LPS/IFN-γ–stimulated MoMFs (M(LPS/IFNγ)) or IL-4–stimulated MoMFs (M(IL4)). Relative *Nrf2* mRNA expression. Data are presented as mean ± SEM (Kruskal–Wallis test, **P* < 0.05, *n* = 5, biological replicates for each group).
n Bone marrow–derived monocytes treated with or without 2 mM NaNO_3_, and then transfected with *Ctsl* overexpression plasmid (OE) or empty vector (EV) *in vitro*, induced to differentiate into MoMFs, polarized into LPS/IFN-γ–stimulated MoMFs (M(LPS/IFNγ)) or IL-4–stimulated MoMFs (M(IL4)). Relative *Nrf2* mRNA expression. Data are presented as mean ± SEM (Kruskal–Wallis test, **P* < 0.05, ***P* < 0.01, *n* = 5, biological replicates for each group).

**o-q** Fluorescent multiplex immunohistochemistry images on liver tissue of CtsL^+^ and Nrf2^+^ macrophages and semi quantitative analysis. Data are presented as mean ± SEM (One-way ANOVA with a post hoc test, **P* < 0.05, *n* = 3, biological replicates for each group).

Supplementary Fig.9.

**Supplementary Fig.9 Supplementary of transfection efficiency in human macrophages and alterations under free fatty acids (FFA).**

a Mean fluorescence intensity (MFI, transfection efficiency) after *SLC17A5* (with FAM) or scrambled siRNA transfected. Data are presented as mean ± SEM (Student’s *t* test, ****P* < 0.001, *n* = 6, biological replicates for each group).
b Mean fluorescence intensity (MFI, transfection efficiency) after *CTSL* overexpression and EV (no GFP) transfected. Data are presented as mean ± SEM (Kruskal–Wallis test, ****P* < 0.001, *n* =6, biological replicates for each group).

**c-k** Human macrophage cultures with FFA, followed by nitrate administration, and then polarized into LPS/IFN-γ–stimulated MoMFs (M(LPS/IFNγ)) or IL-4–stimulated MoMFs (M(IL4)).

c Schematic overview of experiment.
d Proportion of CD11C^+^ cells in M(LPS/IFNγ). Data are presented as mean ± SEM (One-way ANOVA with a post hoc test, ***P* < 0.01, *n* = 5, biological replicates for each group).
e Apoptosis (Annexin V^+^ cells) in M(LPS/IFNγ). Data are presented as mean ± SEM (One-way ANOVA with a post hoc test, ****P* < 0.001, *n* = 5, biological replicates for each group).
f Relative *CTSL* mRNA expression in M(LPS/IFNγ). Data are presented as mean ± SEM (One-way ANOVA with a post hoc test, ***P* < 0.01, *n* = 5, biological replicates for each group).
g Relative *NRF2* mRNA expression in M(LPS/IFNγ). Data are presented as mean ± SEM (Kruskal–Wallis test, **P* < 0.05, *n* = 5, biological replicates for each group).
h Proportion of CD206^+^ cells in M(IL4). Data are presented as mean ± SEM (One-way ANOVA with a post hoc test, ****P* < 0.001, *n* = 5, biological replicates for each group).
i Apoptosis (Annexin V^+^ cells) in M(IL4). Data are presented as mean ± SEM (One-way ANOVA with a post hoc test, ****P* < 0.001, *n* = 5, biological replicates for each group).
j Relative *CTSL* mRNA expression in M(IL4). Data are presented as mean ± SEM (Kruskal–Wallis test, **P* < 0.05, ***P* < 0.01, *n* = 5, biological replicates for each group).
k Relative *NRF2* mRNA expression in M(IL4). Data are presented as mean ± SEM (Kruskal–Wallis test, **P* < 0.05, ***P* < 0.01, *n* = 5, biological replicates for each group).

Supplementary Table 1.

Supplementary Table 1 Gene primer sequences.

| **Gene Name** | **Species** | **Forward primer (5′-3′)** | **Reverse primer (5′-3′)** |
| --- | --- | --- | --- |
| *Acc1* | Mouse | AAGTCCTTGGTCGGGAAGTATACA | ACTCCCTCAAAGTCATCACAAACA |
| *Acc2* | Mouse | GAAGCCACAGTGAAATCTCG | GATGGTTTGGCCTTTCACAT |
| *Acta2* | Mouse | ATCGTCCACCGCAAATGC | AAGGAACTGGAGGCGCTG |
| *Arg1* | Mouse | TGACTGAAGTAGACAAGCTGGGGAT | CGACATCAAAGCTCAGGTGAATCGG |
| *Bcl2* | Mouse | GGAAGGTAGTGTGTGTGG | ACTCCACTCTCTGGGTTCTTGG |
| *Bclxl* | Mouse | AACATCCCAGCTTCACATAACCCC | GCGACCCCAGTTTACTCCATCC |
| *Cd206* | Mouse | CTCAACCCAAGGGCTCTTCTAA | AGGTGGCCTCTTGAGGTATGTG |
| *Cd36* | Mouse | ATGGGCTGTGATCGGAACTG | GTCTTCCCAATAAGCATGTCTCC |
| *Col1a1* | Mouse | CTGCTGGCAAAGATGGAGA | ACCAGGAAGACCCTGGAATC |
| *Col3a1* | Mouse | CAAATGGCATCCCAGGAG | CATCTCGGCCAGGTTCTC |
| *Cpt1a* | Mouse | CTCCGCCTGAGCCATGAAG | CACCAGTGATGATGCCATTCT |
| *Cpt1b* | Mouse | ATGTATCGCCGCAAACTGGACC | CTCTGAGAGGTGCTGTAGCAAG |
| *Cpt1c* | Mouse | TCTTCACTGAGTTCCGATGGG | ACGCCAGAGATGCCTTTTCC |
| *Cpt2* | Mouse | TTCACCACTAAGAACGGTTCG | CTCCAAGGATGACACTGATTTCA |
| *Ctsl* | Mouse | TCTGTTGCTATGGACGCAAG | CCGGTCTTTGGCTATTTTGA |
| *Fabp4* | Mouse | TGAAATCACCGCAGACGACAGG | GCTTGTCACCATCTCGTTTTCTC |
| *Fas* | Mouse | GAGGCCCATTTTGCTGTCAA | CAGGGTGCAGTTTGTTTCCA |
| *Fasn* | Mouse | GATCCTGGAACGAGAACACGAT | GAGACGTGTCACTCCTGGACTTG |
| *Gapdh* | Mouse | GGTGAAGGTCGGTGTGAACG | CTCGCTCCTGGAAGATGGTG |
| *Ifng* | Mouse | GGCCATCAGCAACAACATAAGCGT | TGGGTTGTTGACCTCAAACTTGGC |
| *Il10* | Mouse | AGAAAAGAGAGCTCCATCATGC | TTATTGTCTTCCCGGCTGTACT |
| *Il12b* | Mouse | TCATCAGGGACATCATCAAAC | TGAGGGAGAAGTAGGAATGGG |
| *Il13* | Mouse | GCAGCATGGTATGGAGTGTG | TGGCGAAACAGTTGCTTTGT |
| *Il1b* | Mouse | CCCTGCAGCTGGAGAGTGTGGA | TGTGCTCTGCTTGTGAGGTGCTG |
| *Il6* | Mouse | CTTCCATCCAGTTGCCTTCTTG | AATTAAGCCTCCGACTTGTGAAG |
| *Inos* | Mouse | ATCTTTGCCACCAAGATGGCCTGG | TTCCTGTGCTGTGCTACAGTTCCG |
| *Keap1* | Mouse | CAGCTACACACTAGAGGATCACA | GTGGATGCCTTCGATGGACA |
| *Lpl* | Mouse | CATCGAGAGGATCCGAGTGAA | TGCTGAGTCCTTTCCCTTCTG |
| *Nrf2* | Mouse | AGGACATGGAGCAAGTTTGG | TCCTCAAAACCATGAAGGAA |
| *Pik3cd* | Mouse | CCAGCAGGGGAAGACTAACA | CAGTGCCAGAGAGTCCTTGA |
| *PGC1α* | Mouse | AGGGTGCATGGCAGTTGT | GCTTCTGCGAGAGCTGAGTT |
| *Rel* | Mouse | AGACTGCGACCTCAATGTGG | GCACGGTTGTCATAAATTGGGTT |
| *Scd1* | Mouse | ACTGTGGAGACGTGTTCTGGA | ACGGGTGTCTGGTAGACCTC |
| *Slc17a5* | Mouse | TGCGTTCCTAACCATATCCAC | GACAGTCTGCCATTCCCTAAT |
| *Tgfb1* | Mouse | GAGGTCACCCGCGTGCTA | TGTGTGAGATGTCTTTGGTTTTCTC |
| *Tnf* | Mouse | TCCCAGGTTCTCTTCAAGGGA | GGTGAGGAGCACGTAGTCGG |
| *Trp53* | Mouse | ACAGTCGGATATCAGCCTCG | GCTTCACTTGGGCCTTCAAA |
| *ARG1* | Human | TGGACAGACTAGGAATTGGCA | CCAGTCCGTCAACATCAAAACT |
| *CD206* | Human | GGGTTGCTATCACTCTCTATGC | TTTCTTGTCTGTTGCCGTAGTT |
| *CTSL* | Human | AAACTGGGAGGCTTATCTCACT | GCATAATCCATTAGGCCACCAT |
| *GAPDH* | Human | GGAGCGAGATCCCTCCAAAAT | GGCTGTTGTCATACTTCTCATGG |
| *IL10* | Human | TCAAGGCGCATGTGAACTCC | GATGTCAAACTCACTCATGGCT |
| *IL12b* | Human | TCTTCACGGACAAGACCTCAG | CTAACTGCAGGGCACAGATG |
| *INOS* | Human | TCATCCGCTATGCTGGCTAC | CCCGAAACCACTCGTATTTGG |
| *NRF2* | Human | TCAGCGACGGAAAGAGTATGA | CCACTGGTTTCTGACTGGATGT |
| *SLC17A5* | Human | GCTCTGCTCGTTACAACTTAGC | GAGTTTCTGCATCCCATTGGTA |
| *TNF* | Human | GAGGCCAAGCCCTGGTATG | CGGGCCGATTGATCTCAGC |
